# Transcriptome-Guided Mining of Genes Involved in Crocin Biosynthesis

**DOI:** 10.3389/fpls.2017.00518

**Published:** 2017-04-11

**Authors:** Aijia Ji, Jing Jia, Zhichao Xu, Ying Li, Wu Bi, Fengming Ren, Chunnian He, Jie Liu, Kaizhi Hu, Jingyuan Song

**Affiliations:** ^1^Institute of Medicinal Plant Development, Chinese Academy of Medical Sciences and Peking Union Medical CollegeBeijing, China; ^2^Chongqing Institute of Medicinal Plant CultivationChongqing, China

**Keywords:** transcriptome analysis, crocin biosynthesis, *Gardenia jasminoides*, CCD4a, fruit-specific expression

## Abstract

*Gardenia jasminoides* is used in traditional Chinese medicine and has drawn attention as a rich source of crocin, a compound with reported activity against various cancers, depression and cardiovascular disease. However, genetic information on the crocin biosynthetic pathway of *G. jasminoides* is scarce. In this study, we performed a transcriptome analysis of the leaves, green fruits, and red fruits of *G. jasminoides* to identify and predict the genes that encode key enzymes responsible for crocin production, compared with *Crocus sativus*. Twenty-seven putative pathway genes were specifically expressed in the fruits, consistent with the distribution of crocin in *G. jasminoides*. Twenty-four of these genes were reported for the first time, and a novel *CCD4a* gene was predicted that encodes carotenoid cleavage dioxygenase leading to crocin synthesis, in contrast to CCD2 of *C. sativus*. In addition, 6 other candidate genes (*ALDH12, ALDH14, UGT94U1, UGT86D1, UGT71H4*, and *UGT85K18*) were predicted to be involved in crocin biosynthesis following phylogenetic analysis and different gene expression profiles. Identifying the genes that encode key enzymes should help elucidate the crocin biosynthesis pathway.

## Introduction

**Figure 1:**
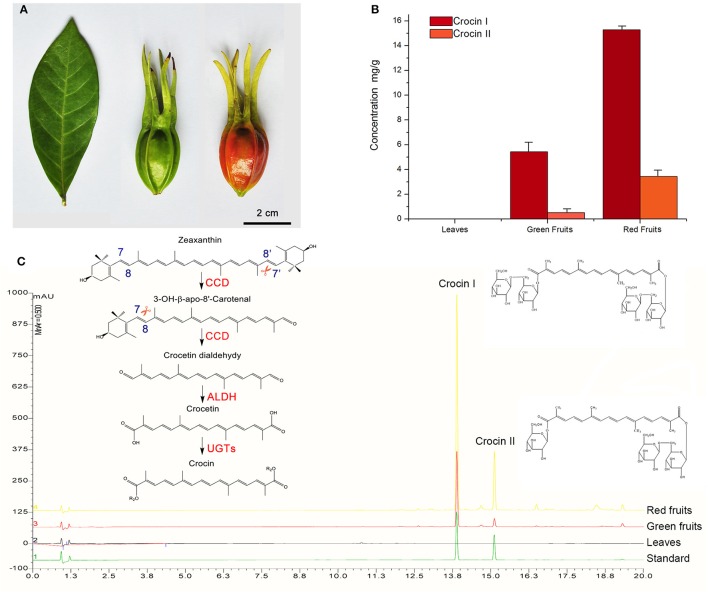
**Quantitative analysis of crocin in various *G. jasmonoides* tissues. (A)** The organs (leaves, green fruits and red fruits) used in this study. **(B)** Corresponding histograms indicate the difference in concentration of crocin among the different organs. **(C)** UPLC chromatograms of the crocin content in the different organs and the proposed crocin biosynthesis pathway. Each sample was analyzed with three replicates. Photodiode array detection at a wavelength of 440 nm was used.

Crocin has received considerable attention in Asia and Europe as a therapeutic agent for the treatment of various diseases. Studies have demonstrated that the growth of cancer cells, such as breast, colorectal, and prostate cancer cells, are altered by crocin via the inhibition of replication (Bhandari, 2015). Crocin has also been shown to significantly improve memory, to have an antidepressant effect in mice (Wang et al., 2010; Hosseinzadeh et al., 2012), and to elicit protective effects against cardiovascular disease (Razavi et al., 2013). Crocin has been found in the stigmas of *Crocus sativus* L. and the fruit of *Gardenia jasminoides* (Sheu and Hsin, 1998; Frusciante et al., 2014). Because of the pharmacological benefits and complex harvesting process of *C. sativus*, the retail price of the red stigmas of *C. sativus*, which is called “red gold,” ranges between 2,000 and 7,000 £/kg (Frusciante et al., 2014). However, the fruit of *G. jasminoides* used in Chinese medicine have a low price because of their abundance and ease of cultivation (Figure 1A).

Recently, determining the pathway of crocin biosynthesis in plants has received increasing attention because of its high medicinal and commercial value (Figure 1C). The crocin biosynthesis pathway in *C. sativus* includes the upstream methylerythritol phosphate (MEP) pathway from pyruvate/glyceraldehyde 3-phosphate to geranylgeranyl pyrophosphate (GGPP), the midstream carotenoid pathway from GGPP to zeaxanthin, and the downstream crocin pathway from zeaxanthin to crocin. In the crocin pathway, crocin is generated from the oxidative cleavage of zeaxanthin at the 7,8/7′,8′ positions by carotenoid cleavage dioxygenase (CCD). The cleavage product crocetin dialdehyde is further dehydrogenated and glycosylated to crocin by an aldehyde dehydrogenase (ALDH) and glucosyltransferases (UGTs) (Frusciante et al., 2014). Although CCD, ALDH, and UGT enzymes that may be responsible for crocin biosynthesis are known, the exact genes in *C. sativus* that encode these enzymes have seldom been subjected to functional analyses. The genes of the MEP and carotenoid pathways have been identified in many plants Nisar et al., 2015; Xu et al., 2015; Xu H. et al., 2016; Xu Z.-C. et al., 2016; however, few of these genes have been functionally analyzed in *C. sativus* (Ahrazem et al., 2009), and most researchers have explored the functions of the CCD, ALDH and UGT enzymes, such as CsCCD1a, CsCCD1b, CsCCD2, CaCCD2, CsCCD4a, CsCCD4b, CsCCD4c, CsUGT2, UGT707B1, and CsGT45 (Moraga et al., 2004, 2009; Rubio et al., 2008; Nagatoshi et al., 2012; Trapero et al., 2012; Frusciante et al., 2014; Ahrazem et al., 2016). However, only 3 of these enzymes are associated with crocin biosynthesis. One carotenoid cleavage dioxygenase (CsCCD2) of *C. sativus* was identified through 454 sequencing of transcriptomes, and the expression of the *CsCCD2* gene was consistent with the accumulation of crocin (Frusciante et al., 2014). After performing a functional characterization, CsCCD2 was found to catalyze the conversion of zeaxanthin into crocetin dialdehyde via cleavage at the 7,8 and 7′,8′ positions. In *Crocus ancyrensis*, the expression of CaCCD2 was also correlated with the accumulation of crocin, and CaCCD2 was found to catalyze crocetin formation in bacteria and rice (Ahrazem et al., 2016). Moreover, UGTCs2 was identified in *C. sativus*, and an *in vitro* experiment showed that crocetin can be glycosylated by UGTCs2 (Moraga et al., 2004).

In *Gardenia jasminoides*, two UGT enzymes (UGT75L6 and UGT94E5) were shown to glycosylate crocetin in the last step of the crocin pathway (Nagatoshi et al., 2012). Previous studies revealed that carotenoid accumulation in a specific organ is correlated with the up-regulation of genes that encode key enzymes (Botella-Pavía and Rodríguez-Concepción, 2006). The expression levels of *CsCCD2* and *CaCCD2* were both correlated with the accumulation of crocin. However, the study by Nagatoshi et al. indicated that the expression of *UGT75L6* and *UGT94E5* did not differ among various organs, and the authors suggested that these two UGTs were not rate-limiting enzymes for crocin biosynthesis. Considering the correlation between secondary metabolites levels and expression of the putative pathway genes (Ma et al., 2015; Xu et al., 2015; Xu Z. et al., 2016), we speculated that other UGTs may be responsible for crocin biosynthesis in *G. jasminoides*.

In this study, we propose that *G. jasminoides* has the potential for use as an alternative plant for research into the crocin biosynthesis pathway. *G. jasminoides* has been cultivated for at least a thousand years in China for use as an ornamental plant and for traditional medicine. In the mid-eighteenth century, *G. jasminoides* was introduced to English gardens, and it is now distributed widely around the world (Liu and Yin, 2015). Moreover, studies on its chemicals and pharmacological effects have provided a foundation for further analyses of the crocin biosynthesis pathway (Sheng et al., 2006; Chen et al., 2010). Most importantly, compared with *C. sativus*, which specifically accumulates crocin in the stigma, obtaining the fruit material of *G. jasminoides* for research on crocin biosynthesis is much easier. *G. jasminoides* reportedly accumulates crocin specifically in its fruit, and the content increases during fruit development. The contents of crocin I and crocin II in the pulps are up to 28 mg g^−1^ (He et al., 2006). Here, a comparative transcriptome analysis was performed for the leaves, green fruit and red fruit of *G. jasminoides* and candidate crocin biosynthetic genes were screened based on the expression of different genes. Furthermore, quantitative real-time PCR (qRT-PCR) analysis, phylogenetic analysis and quantitative analysis of crocin supported the role of the candidate genes in crocin biosynthesis.

## Materials and methods

### Plant material and RNA extraction

In this study, *G. jasminoides* Ellis, which was cultivated at the medicinal garden at the Chongqing Institute of Medicinal Plant Cultivation (China), was used. The leaves, green fruits, and red fruits were collected from three trees, respectively; and immediately frozen in liquid nitrogen for RNA isolation. Total RNA was isolated from each sample using an RNAprep Pure Plant Kit (Tiangen Biotech., Beijing, China) according to the manufacturer's instructions. The quality and quantity of the RNA were analyzed by electrophoresis and a NanoDrop 2000C spectrophotometer. The RNA Integrity Number (RIN) was determined using the BioAnalyzer 2100 (Agilent Technologies) to ensure that the RIN values were >6.5.

### RNA library construction and sequencing

The cDNA libraries were created according to the manufacturer's protocol (NEBNext® Ultra™ RNA Library Prep Kit for Illumina®). First strand cDNA was synthesized using ProtoScript II Reverse Transcriptase. After the synthesis of the second-strand cDNA using the Second Strand Synthesis Enzyme Mix, the end repairing, 5′ phosphorylation and dA-tailing were performed in one reaction. Approximately 250 bp cDNA fragments were selected using AxyPrep Mag PCR Clean-up (Axygen) for the library construction. The libraries with different indexes were paired-end sequenced using the Illumina HiSeq 2000 instrument.

### Transcriptome data processing and assembly

The raw reads with adaptor and poly-N sequences were removed, and low quality reads (*Q*-value < 10 in more than 50% of the bases) were also removed. Clean data with high sequence quality were used in all of the downstream analyses. Transcriptome assembly was conducted using Trinity software with the minimum K-mer value set to 1. After removing the redundancy with cd-hit-est software (Li and Godzik, 2006), unigenes were obtained. For the unigene annotation, the unigenes were searched against the NCBI protein sequence database using BLASTp and a cutoff *E*-value of 10^−5^. The unigenes were also annotated with the following databases: Pfam (Protein family), KO (KEGG Ortholog database), and GO (Gene Ontology). Benchmarking Universal Single-Copy Orthologs (BUSCO) software was used to assess transcriptome assembly and annotation completeness.

### GO and KEGG pathway enrichment analysis

The Gene Ontology annotations were assigned based on their similarity to the *A. thaliana* proteomic sequences (TAIR10). The transcripts were classified into 45 GO categories under the major categories of Cellular Component, Molecular Function and Biological Process. The transcriptome sequences were BLAST against KEGG GENES databases, and the KEGG classification was analyzed according to the KO number in the annotation results.

### Differential expression analysis

The gene expression levels were estimated using RSEM software. The DEGs (differentially expressed genes) analysis among the different organs was performed using DESeq Bioconductor package (1.14.0). The genes with absolute values of log2 fold changes >= 1 (FDR value < 0.001) were identified as DEGs.

### qRT-PCR analysis

Total RNA was extracted from the red fruits, green fruits and leaves using an RNAprep Pure Plant Kit (Tiangen Biotech, Beijing, China) according to the manufacturer's instructions. After analyzing the quality and integrity of the RNA, first-strand cDNA was synthesized using a PrimeScript™ II 1st Strand cDNA Synthesis Kit (Takara, Beijing, China). The specific primers (130–180 bp) were designed using Primer Premier 6.0 (Supplemental Table 1). The qRT-PCR experiment was performed on an ABI 7500 instrument and the reagents and methods were identical to that of our previous study (Luo et al., 2014). *Actin* and *GAPDH* were used as internal controls. qRT-PCR analysis was performed with three biological and technical replicates. To detect expression differences of the candidate CCD, ALDH, and UGT genes among various organs, one-way ANOVA test was performed by IBM SPSS 20 software. *P* < 0.01 was considered highly significant.

### Phylogenetic and conserved motif analyses

The CCD, ALDH, and UGT protein sequences of *G. jasminoides* and other plants were aligned by MEGA 5.0 software. A neighbor-joining (NJ) tree was constructed using a Poisson model with 1,000 bootstrap replicates. The protein sequences included CCD1, CCD4a, NCED1, NCED2, ALDH12, ALDH14, UGT60, UGT67, UGT86, and UGT89 from this study; AtCCD1, AtCCD4, AtNCED3, AtNCED5, AtNCED6, AtCCD7, AtCCD8, AtALDH2C4, AtALDH2B7 UGT73C6, and UGT73B2 from *A. thaliana* (Skibbe et al., 2002; Jones et al., 2003; Nair et al., 2004; Auldridge et al., 2006; Kim et al., 2006; Alder et al., 2012; Frey et al., 2012; Gonzalez-Jorge et al., 2013); CsCCD2, CsGT45, and UGTCs2 from *C. sativus* (Moraga et al., 2004; Frusciante et al., 2014); MtCCD1 and UGT73K1 from *Medicago truncatula* (Achnine et al., 2005; Floss et al., 2008; Moraga et al., 2009); CmCCD1 from *Cucumis melo* (Ibdah et al., 2006); CmCCD4a from *C. morifolium* (Ohmiya et al., 2006); CitCCD4 from *C. unshiu* (Ma et al., 2013; Rodrigo et al., 2013); CaALDH1 from *C. annuum* (Kim and Hwang, 2015); AaALDH1 from *A. annua* (Teoh et al., 2009); BoBADH from *B. orellana* (Bouvier et al., 2003); REF1 from *B. napus* (Mittasch et al., 2013); Zmrf2 (ALDH2B2) from *Z. mays* (Cui et al., 1996); BALDH from *A. majus* (Long et al., 2009); UGT75L6 and UGT94E5 from *G. jasminoides* (Nagatoshi et al., 2012); VLOGT1, VLOGT2, and VLOGT3 from *Vitis labrusca* (Hall et al., 2011); Gt5GT7 from *Gentiana triflora* (Nakatsuka et al., 2008); UGT1, UGTPg29, and UGTPg45 from *P. ginseng* (Yan et al., 2014; Wang et al., 2015); CrUGT8 from *Catharanthus roseus* (Asada et al., 2013); AdGT4 from *Actinidia deliciosa* (Yauk et al., 2014); GAME2 from *Solanum lycopersicum* (Itkin et al., 2013); ZOG1 from *Phaseolus lunatus* (Martin et al., 1999b); and ZOX1 from *Phaseolus vulgaris* (Martin et al., 1999a). Conserved motifs in CCDs, ALDHs and UGTs were detected using motif based sequence analysis tool MEME (Suite version 4.11.2). The Mw and p*I* of the proteins were predicted using online bioinformatics tool ExPASy proteomics (http://web.expasy.org/protparam/).

### Crocin identification by UPLC

Crocin I and crocin II were purchased from the National Institutes for Food and Drug Control (China). Acetonitrile and methanol were of chromatographic purity, and all of the other solvents were analytically pure. The red fruits, green fruits and leaves with three repetitions were freeze dried and then ground to a powder for each sample. The powder of each sample (0.25 g) was accurately weighed and extracted with 50% methanol to a final volume of 25 mL. After treatment with an ultrasonic instrument for 45 min, 50% methanol was added to complement the weight loss and the sample was then filtered. The filtrate was further passed through a 0.22 μm membrane filter to yield the test sample solution. Moreover, the mixed standard solution that included 0.035 mg/ml crocin I and 0.015 mg/ml crocin II was prepared by dissolving the sample in methanol. The chromatographic analysis was performed on a DIONEX Ultimate 3,000 UPLC system with a DAD detector (Thermo Fisher Scientific, USA). The UPLC separation was performed on Waters BEH-C18 column (100 × 2.1 mm, 1.7 μm), operated at 35°C. The mobile phase was acetonitrile (A) and H_2_O (B) with the flow rate of 0.3 mL/min. The injection volume was 2 μL and the mobile phase gradient was as follows: 0–6 min, 5–12% A; 6–25 min, 12–48% A. The detection wavelength of crocins was 440 nm.

## Results

### Sequencing, *de novo* assembly and functional annotation

In this study, 45,224,600, 46,735,458, and 60,458,180 raw Illumina cDNA sequencing reads were obtained from RNA extracted from leaves, green fruits and red fruits, respectively, of *G. jasmonoides* (Figure 1A). After data filtering, a total of 147,167,330 clean reads of high quality were generated for further analysis (Table 1). The summary of the RNA-Seq data for each sample is illustrated in Supplemental Table 2. The clean reads were assembled into 156,658 contigs using Trinity software. After removing the redundant contigs, 141,665 unigenes were identified, and 58,702 coding sequences (CDSs) longer than 300 bp were detected using a BLAST search against protein databases (Table 1). The size distribution of the contigs, unigenes and CDS is presented in Supplemental Figure 1. In addition, we applied the BUSCO software to assess the assembly and annotation completeness. As 1,440 single copy orthologs for plants were used, our assembly is 84.7% complete, while 6.7% of contigs are fragmented and 8.6% are missing.

**Table 1:** **Summary of the illumina paired-end sequencing and assembly for *Gardenia jasminoides***.

**Item**	**Number**	**Length (bp)**
Total clean reads	147,167,330	14,370,474,299
Contigs	156,658	141,953,000
Average length of contigs		906
Contig size N50		1,632
Unigenes	141,665	122,469,676
Average length of unigenes		864
Unigene size N50		1,574
CDS	58,702	51,532,023
Average length of CDS		878

The protein sequences were aligned to the Uniprot, Pfam, KEGG, and GO databases for annotation. A total of 52,816 CDSs (90%) were annotated, including 37,961 (64.7%) in Uniprot, 41,907 (71.4%) in Pfam, 11,375 (19.4%) in KEGG and 45,715 (77.9%) in the Gene Ontology (GO) database (Supplemental Table 3). All of the unigenes were functionally categorized by a GO analysis based on their sequence similarity with *Arabidopsis thaliana* (Supplemental Figure 2A). A total of 52,296 unigenes were characterized into the following three categories: “Molecular Function” (47,272), “Biological Process” (48,695), and “Cellular Component” (49,743). In addition, a KEGG enrichment analysis was performed to detect the genes in the active compound metabolic pathway (Supplemental Figure 2B). A total of 24,991 unigenes matched to the KEGG database and were assigned to 5 major pathways.

### Genes encoding key enzymes in the MEP, carotenoid and crocin biosynthetic pathways

We analyzed the differentially expressed genes (DEGs) among the leaves, green fruits and red fruits. Compared with the leaves, the expression levels of 1,958 and 995 genes were up- and down-regulated in the green fruits, respectively, whereas the expression levels of 3,761 and 967 genes were up- and down-regulated in the red fruits, respectively. In addition, 282 and 421 genes were up- and down-regulated in the red fruits relative to green fruits, respectively (Supplemental Figure 3). All of the DEGs are summarized in Supplemental Table 4.

Twenty-seven genes involved in crocin biosynthesis, including the upstream MEP pathway, the midstream carotenoid pathway, and the downstream crocin pathway, were also identified and selected. Among them, we detected 17 unigenes encoding 9 enzymes in the pathways involved in the synthesis of geranylgeranyl diphosphate (GGPP), a late carotenoid precursor. As shown in Supplemental Figure 4, some enzymes were represented by more than one unigene. Four unigenes were predicted to encode 1-deoxy-D-xylulose-5-phosphate synthase (DXS), two unigenes were predicted to encode isopentenyl-diphosphate delta-isomerase (IDI), and five unigenes were predicted to encode geranylgeranyl diphosphate synthase (GGPPS). Based on the gene expression profiles, five genes (*DXS1, DXR, IDI1, GGPPS2*, and *GGPPS4*) were more highly expressed in the red fruit than the green fruit and leaves (Supplemental Figure 4). The expression of these genes is consistent with the distribution of crocin; therefore, these five genes may be responsible for crocin biosynthesis.

**Figure 2:**
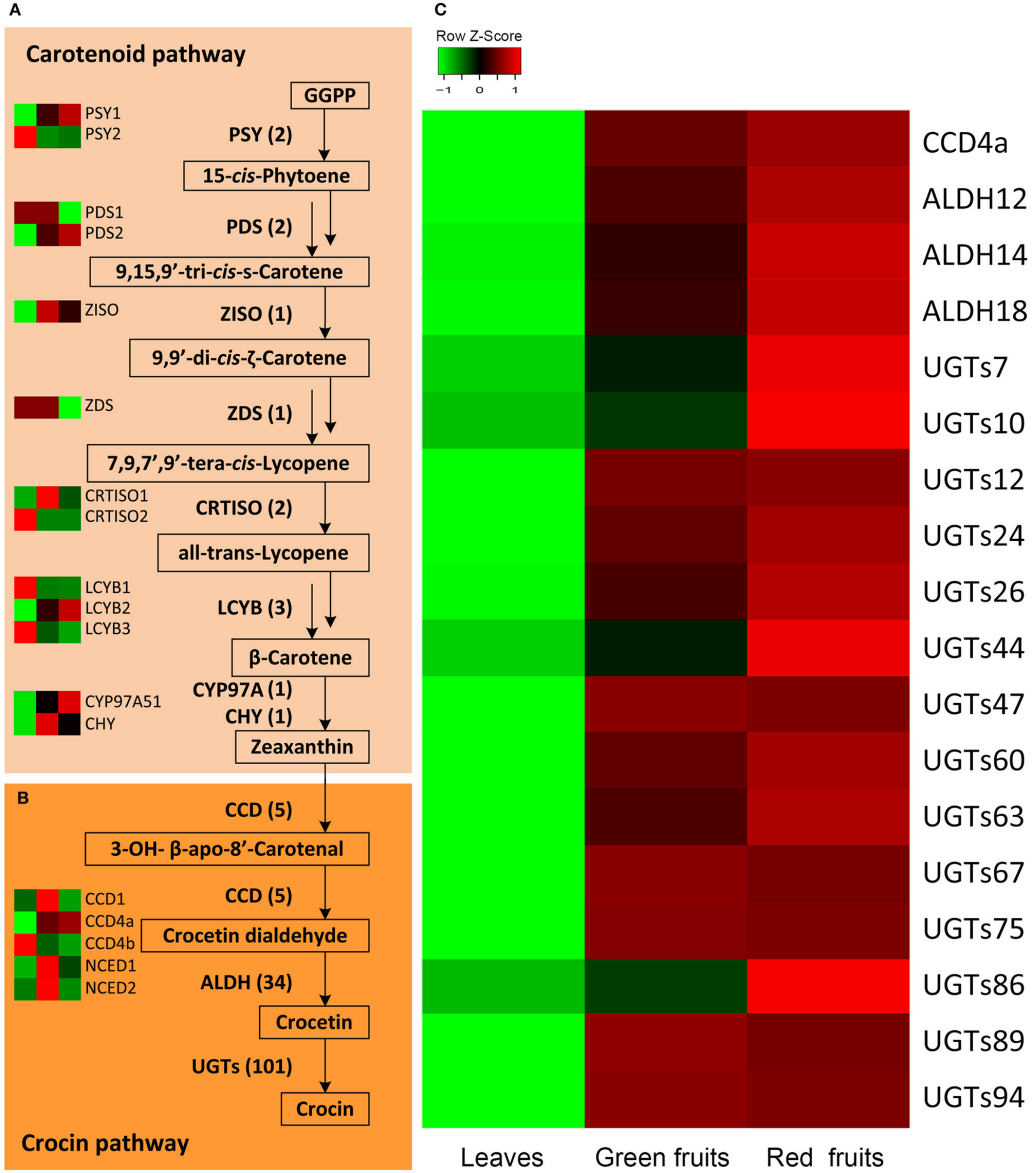
**Expression profile of candidate *G. jasmonoides* genes for crocin biosynthesis. (A)** The proposed carotenoid biosynthetic pathway of *G. jasmonoides*. **(B)** The proposed crocin biosynthetic pathway in *G. jasmonoides*. **(C)** The expression of candidate CCDs, ALDHs, and UGTs from differential expression analysis.

In the carotenoid pathway, 13 genes encoding eight enzymes were identified in our transcriptome data (Figure 2A). The expression levels of *phytoene synthase 1* (*PSY1*), *phytoene desaturase 2* (*PDS2*), *lycopene* β*-cyclase 2* (*LCYB2*), and *cytochrome P450-type monooxygenase 97A51* (*CYP97A51*) were all increased as the fruit ripened, and the fragments per kilobase of transcript per million mapped reads (FPKM) value of *PSY1* reached 795.6 in the red fruits (Supplemental Table 5). Moreover, *PDS2* presented a nearly 7-fold change in the red fruits compared with that in the leaves. However, ζ*-carotene isomerase* (*Z-ISO*), and *carotenoid* β*-hydroxylase* (*CHY1*) were both expressed at higher levels in the green fruits and red fruits compared with the leaves. The gene expression of ζ*-carotene desaturase* (*ZDS*) and *carotenoid isomerase* (*CRTISO*) did not change in all of the samples. Therefore, *PSY1, PDS2, LCYB2*, and *CYP97A51* are likely to be involved in crocin biosynthesis.

The crocin biosynthetic pathway includes CCD, ALDH, and UGT enzymes (Figure 2). In this study, a total of 140 unigenes were identified, including 5 *CCDs*, 34 *ALDHs* and 101 *UGTs* (Supplemental Figure 5 and Supplemental Table 5). The plant CCD family is divided into five subfamilies: CCD1, CCD4, CCD7, CCD8, and NCED (nine-cis-epoxy-carotenoid dioxygenase; Tan et al., 2003). Five CCD members from the transcriptome data of *G. jasminoides* were named CCD1, CCD4a, CCD4b, NCED1, and NCED2 based on their protein sequence similarity with *A. thaliana*. Compared with the different expression of CCD genes among the various organs, CCD4a demonstrated significant and fruit-specific expression, and the FPKM values in the leaves, green fruit and red fruit were 0.5, 1283.3, and 1463.0, respectively, implying a possible functional link to crocin biosynthesis. However, *NCED1* was only slightly expressed in the green fruit, and it was not expressed in the leaves and red fruit. *NCED2* was also expressed at low levels in all of the organs, and the expression level in the green fruit was higher than that in the other two organs. In addition, the expression of *CCD1* did not change among the various organs, whereas *CCD4b* had the highest expression in the leaves. This suggests that *CCD4a* is involved in crocin biosynthesis. ALDHs can catalyze the conversion of crocetin dialdehyde to crocetin (Figure 1). A transcriptome-wide analysis identified 34 *ALDHs*, and 3 *ALDHs* (*ALDH12, ALDH14*, and *ALDH18*) were highly expressed in the fruits (FPKM > 10). Similarly, the gene expression of 14 UGTs was higher in the fruits than in the leaves. Among them, 6 UGTs have significant and fruit-specific expression, and their FPKM values in the fruit reached over 100 (Supplemental Table 5). *CCD4a*, 3 *ALDHs*, and 14 *UGTs* were selected for further analysis. The summary of the 27 selected genes is illustrated in Supplemental Table 6.

### Expression and phylogenetic analysis candidate CCD, ALDH, and UGT genes

**Figure 3:**
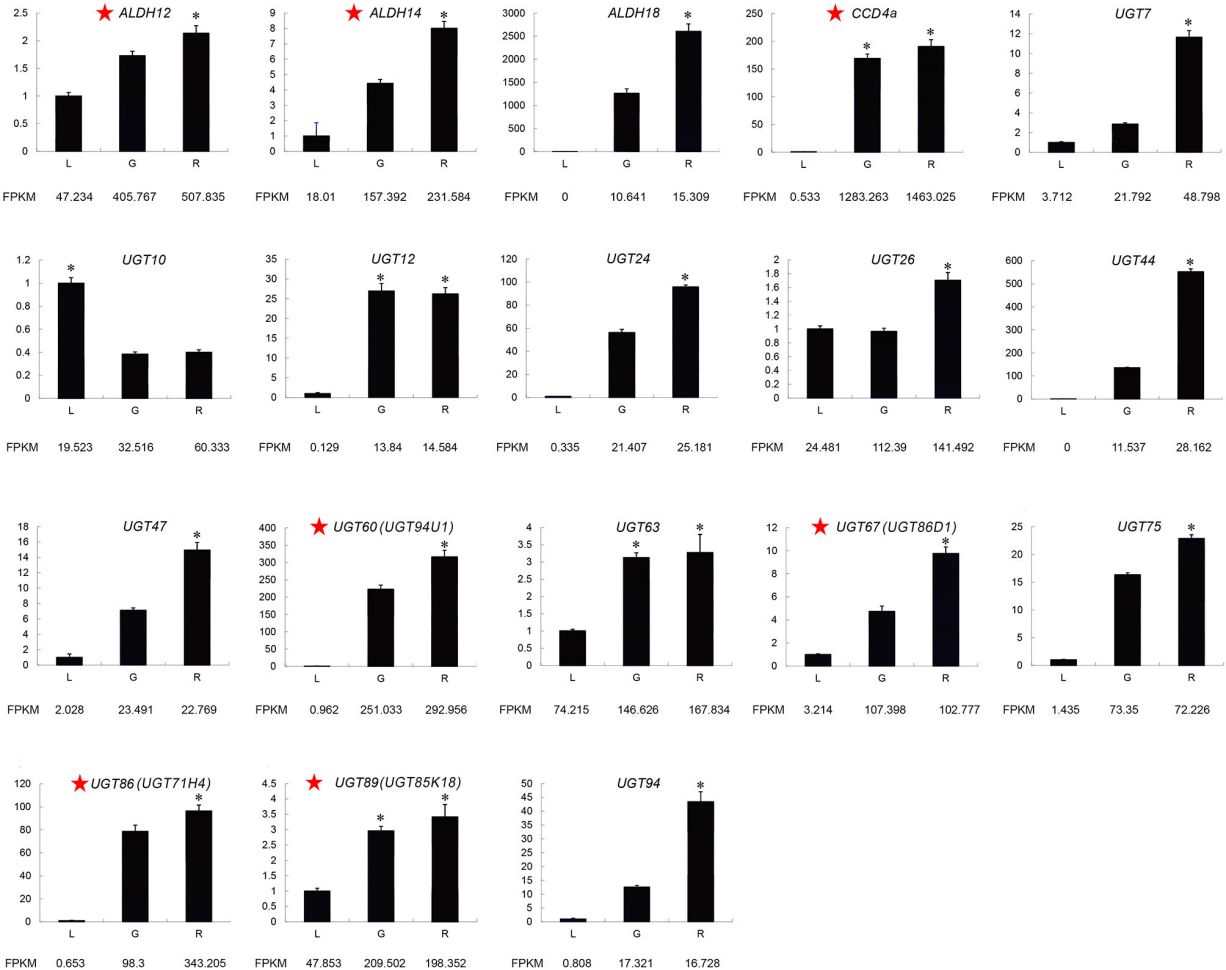
**Expression of candidate crocin biosynthesis genes related to crocin biosynthesis determined by qRT-PCR**. The characters on the X-axis indicate the leaves (L), green fruits (G), and red fruits (R). The Y-axis represents the fold changes in gene expression. The *actin* gene was used as an internal reference. The red star next to gene names represents the most likely to be involved in crocin biosynthesis. One-way ANOVA was performed using IBM SPSS 20 software. Asterisks represents significant differences from this comparison. *P* < 0.01 was considered highly significant.

**Figure 4:**
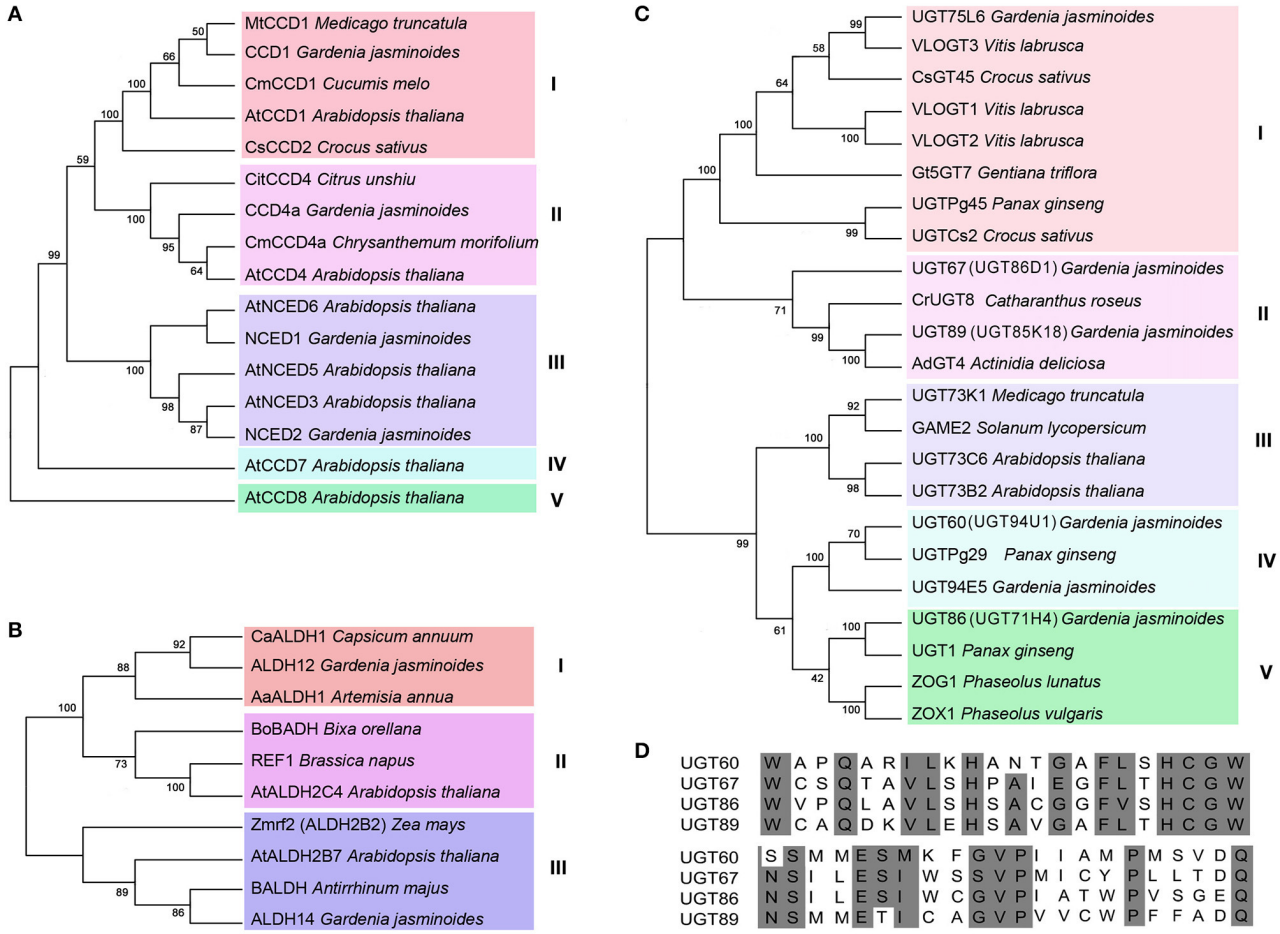
**Phylogenetic and sequence analysis of cadidtate crocin biosynthesis genes**. The Phylogenetic trees of the CCDs **(A)**, ALDHs **(B)**, and UGTs **(C)** between *G. jasminoides* and other plants, and a PSPG box alignment of the candidate UGT proteins **(D)** are shown.

The expression patterns of *CCD4a*, 3 *ALDHs*, and 14 *UGTs* were similar based on different internal control genes (*actin* and *GAPDH*), indicating the reliability of the RNA-Seq results (Figure 3 and Supplemental Figure 6). We found that *ALDH12, ALDH14, ALDH18, CCD4a, UGT7, UGT24, UGT44, UGT47, UGT60* (*UGT94U1*), *UGT67* (*UGT86D1*), *UGT75, UGT86* (*UGT71H4*), *UGT89* (*UGT85K18*), and *UGT94* were more highly expressed in the red fruit than the green fruit and leaves. Moreover, the FPKM values of *ALDH12, ALDH14, CCD4a, UGT60 (UGT94U1), UGT67* (*UGT86D1*), *UGT86* (*UGT71H4*), and *UGT89* (*UGT85K18*) were over 100 in the red fruit. In addition, the UGT proteins related to secondary metabolism were reported to have a PSPG (plant secondary metabolite glycosyltransferase) box. We searched the protein sequences of the 4 UGTs and found that *UGT94U1, UGT86D1, UGT71H4*, and *UGT85K18* all had a PSPG box (Figure 4D). Therefore, we propose that CCD4a, 2 ALDH genes (*ALDH12* and *ALDH14*) and 4 UGT genes (*UGT94U1, UGT86D1, UGT71H4*, and *UGT85K18*) may be related to crocin biosynthesis.

Furthermore, the NJ tree of the CCDs was divided into five groups of subfamilies: CCD1, CCD4, NCED, CCD7, and CCD8 (Figure 4A). We found that *Gardenia* CCD4a was distinct from the CsCCD2 of *C. Sativus* (Frusciante et al., 2014); however, it was a member of group II and closely related to the CitCCD4 enzyme, which can also cleave zeaxanthin at the 7,8 or 7′,8′ positions (Ma et al., 2013; Rodrigo et al., 2013). Thus, based on the analyses of the expression patterns and phylogenetic relationships, *Gardenia* CCD4a may be involved in the oxidative cleavage of the 7,8 and 7′,8′ double bonds of zeaxanthin to give crocetin dialdehyde in the crocin biosynthesis pathway. We performed a bioactivity test of CCD4a in *E. coli*. The plasmid pACCAR25ΔcrtX is capable of producing zeaxanthin. Thus, we co-transformed *E. coli* BL21 (DE3) with pET28a-CCD4a and pACCAR25ΔcrtX, and we named the strain 25ΔcrtX-CCD4a. After IPTG induction, decoloration was observed in strain 25ΔcrtX-CCD4a compared to the control (CK) (Supplemental Figure 7A). HPLC analysis revealed that zeaxanthin accumulation was decreased significantly in 25ΔcrtX-CCD4a (Supplemental Figure 7B). Therefore, we concluded that CCD4a might cleave zeaxanthin at the 7,8 and 7′,8′ positions, thereby leading to crocin synthesis in *G. jasminoides*.

The phylogenetic analysis showed that ALDH12 and ALDH14 belong to the ALDH2C and ALDH2B subfamilies, respectively (Figure 4B). ALDH12 was clustered with CaALDH1 of *Capsicum annuum*, AaALDH1 of *Artemisia annua*, BoBADH of *Bixa orellana*, REF1 of *Brassica napus* and AtALDH2C4 of *A. thaliana*. These proteins are all ALDH2C members. It is worth noting that AaALDH1 and BoBADH control artemisinin and bixin biosynthesis, respectively, and bixin is specifically derived from the carotenoid pathway as well as the crocin. In addition, ALDH14 was grouped with Zmrf2 of *Zea mays*, AtALDH2B7 of *A. thaliana* and BALDH *Antirrhinum majus*, which are all ALDH2B members. Moreover, BALDH has been reported to be involved in benzoic acid biosynthesis. These results indicate that ALDH12 and ALDH14 may be involved in crocin biosynthesis in *G. jasminoides* fruit.

The phylogenetic tree of the UGTs was divided into two clades that contained 5 groups (Figure 4C). UGT86D1 and UGT85K18 were members of group II and grouped with AdGT4 of *A. deliciosa* and CrUGT8 of *C. roseus*. UGT94U1 was closely related to UGT94E5 of *G. jasminoides* and UGTPg29 of *P. ginseng*. Moreover, UGT71H4 was clustered with UGT1 of *P. ginseng* in group V. The UGT proteins that were grouped with the candidate UGTs in this study were involved in terpenoid biosynthesis. Thus, we suggest that UGT94U1, UGT86D1, UGT71H4, and UGT85K18 may be related to the biosynthesis of crocin.

The multiple sequence alignments were performed using the CCDs, ALDHs, and UGTs sequences of phylogenetic trees, respectively. The results indicated that ALDHs proteins among different species were more conserved than CCDs and UGTs (Supplemental Figure 8). The number of amino acids, Mw and p*I* of the proteins were also summarized in Supplemental Table 7. Moreover, diverse conserved motifs in CCDs, ALDHs, and UGTs sequences were identified (Supplemental Figure 9). Five conserved motifs were identified in 10 ALDHs, and 9 of them have all 5 motifs indicating their high conservation (Supplemental Figure 9B). The CCDs have 16 conserved motifs and each subfamily has specific motifs. In CCD4 subfamily, motifs 1–8, 10, 11, and 14 corresponded to all members (Supplemental Figure 9A). Thirteen conserved motifs were detected in the UGT proteins (Supplemental Figure 9C). The motifs distribution of UGT86D1 (UGT67) was most similar to CrUGT8, whereas UGT86D1 (UGT67) got 2 more motifs: motif 8 and 13. UGT85K18(UGT89) shared same motifs with AdGT4 except motif 3. UGT94U1(UGT60) got one more motif than UGT94E5. The motifs distribution of UGT71H4(UGT86) was identical with UGT1.

### Crocin distribution in *G. jasmonoides* tissues

To confirm the correlation between the candidate gene expression and crocin distribution, the crocin content in the green fruits, red fruits and leaves was analyzed by ultra-performance liquid chromatography (UPLC). As presented in Figures 1B,C, the distribution of crocin I and crocin II was fruit specific. After the fruit had ripened, the content of crocin I and crocin II increased, and in the red fruits, the content is almost three and seven times higher relative to the content in the green fruit, respectively. These results suggest that crocin biosynthesis in *G. jasminoides* may be completely conducted in the fruits, and the identification of our 7 candidate genes was confirmed using a co-expression analysis.

## Discussion

We initiated the first transcriptome sequencing of the red fruits, green fruits and leaves of *G. jasminoides* to select genes that encode the key enzymes in the crocin biosynthesis pathway. Twenty-seven pathway genes were expressed at greater levels in the red fruits compared with the green fruits and leaves, and the involvement of 7 of these genes in the crocin pathway was determined by the qRT-PCR and phylogenetic analyses. Moreover, the expression patterns of these 7 genes were consistent with the distribution of crocin in various organs. With the exception of *PSY1* (GenBank No. AEF59491.1), *LCYB2* (GenBank No. AFI09271.1), and *UGT10* (GenBank No. AB555742.1), the 24 candidate genes were described for the first time in this study. Although, the transcriptome analysis of *G. jasminoides* flowers has been previously studied (Tsanakas et al., 2014), the objective of the previous report was the identification of TFs, which may play a role in petal senescence. In this paper, we report work relevant to the biosynthesis of crocin, a compound with important health benefits. This represents a first major step in the identification of the genes involved in crocin biosynthesis.

A novel CCD4 subfamily member that is different from CCD2 of *C. sativus* was most likely to be involved in crocin biosynthesis. Five CCDs, including 1 CCD1, 2 CCD4s, and 2 NCEDs, were identified in our study. CsCCD2 of *C. sativus* and CaCCD2 of *C. ancyrensis* were the enzymes responsible for the first dedicated step leading to crocin biosynthesis starting with zeaxanthin, and their expression pattern was consistent with crocin production (Frusciante et al., 2014; Ahrazem et al., 2016). CCD2 was not observed in our data. The phylogenetic analysis indicated that CsCCD2 was clustered with the CCD1 subfamily members in group I (Figure 4A). CCD1 of *G. jasminoides* was also located in group I, although its expression level did not change in the fruit and leaves. Thus, CCD1 may be not associated with crocin accumulation. Remarkably, CCD4a of *G. jasminoides* was more highly expressed in the red fruit than the green fruit, and it was scarcely expressed in the leaves. In addition, several CCD4s from other plants were also reported to be key determinants of pigmentation. For example, the petal color of *C. morifolium* changes from white to yellow through RNA interference (RNAi) of a *CmCCD4a* gene, which is specifically transcribed in flowers (Ohmiya et al., 2006). The carotenoid level of RNAi lines of potato, in which the *StCCD4* gene is down-regulated, are 2- to 5-fold higher than that of the control (Campbell et al., 2010; Bruno et al., 2015). Moreover, in *A. thaliana*, the expression level of AtCCD4 is inversely correlated with pigmentation, and the loss of AtCCD4 function in *A. thaliana* results in increased β-carotene content (Gonzalez-Jorge et al., 2013). In addition, the CmCCD4a, StCCD4, and AtCCD4 enzymes all cleave their substrates at the 9,10 and 9′,10′ positions (Huang et al., 2009; Bruno et al., 2015). CCD4 appears to act as a negative regulator of carotenoid content and has cleavage position specificity. However, recent studies on a CCD4 from *Citrus unshiu* demonstrated a consistent expression pattern with the accumulation of β-citraurin, and CitCCD4 was found to cleave the 7,8 or 7′,8′ bond of β-cryptoxanthin and zeaxanthin and contributed to the formation of β-citraurin (Ma et al., 2013; Rodrigo et al., 2013). We also characterized the function of CCD4a *in vitro* and the results indicated zeaxanthin can be cleaved by CCD4a. Therefore, we conclude that CCD4a might cleave zeaxanthin at the 7,8 and 7′,8′ positions, thereby leading to crocin synthesis in *G. jasminoides*.

The last step of crocin biosynthesis involves UDP-glucosyltransferases (UGTs), which convert crocetin to crocin. In this study, 101 UGT genes were detected in our RNA-Seq data, and four genes (*UGT94U1, UGT86D1, UGT71H4*, and *UGT85K18*) were proposed as candidate genes in crocin biosynthesis. First, the expression of these genes increased during fruit development, and the lowest expression levels were observed in the leaves. *UGT94U1 and UGT71H4* presented fruit-specific expression levels, implying their potential functions. Second, these UGTs all had a conserved motif called the PSPG box, which is involved in secondary metabolism. Finally, the phylogenetic analysis indicated that although these genes were clustered in different groups, they were all grouped with the UGTs that were functionally characterized as involved in terpenoid biosynthesis. UGT86D1 and UGT85K18 were in group II with members of AdGT4 from *A. deliciosa* and CrUGT8 from *C. roseus* (Asada et al., 2013; Yauk et al., 2014). In *A. deliciosa*, a range of terpenes are glycosylated by AdGT4; and in *C. roseus*, CrUGT8 was identified to be responsible for the formation of secologanin. In addition, UGT94U1 was closely related to UGTPg29 from *Panax ginseng* and UGT94E5 from *G. jasminoides*, which were involved in ginsenoside and crocin biosynthesis, respectively (Nagatoshi et al., 2012; Wang et al., 2015). Moreover, UGT71H4 was also clustered with a UGT from *P. ginseng* called UGTPg1, which was the key to the biosynthesis of compound K, a ginsenoside (Yan et al., 2014). We suggest that UGT94U1, UGT86D1, UGT71H4, and UGT85K18 may function as key enzymes in the biosynthesis of crocin. UGT75L6 and UGT94E5 from *G. jasminoides* were functionally analyzed in a previous study (Nagatoshi et al., 2012). Although they were not included as candidate genes in our study, our transcriptome data identified these genes. The protein sequence of UGT1 has a 92.4% similar identity to UGT75L6, and it was more highly expressed in the leaves than the fruits. In addition, UGT52, which has only one amino acid variation from UGT94E5 (99.8% identity), was highly expressed in the fruits, although the expression level in the fruits was not more than twice that of the leaves. The UGT genes that showed significant or specific expression in the fruits are most likely responsible for crocin biosynthesis. Therefore, a comparative transcriptome analysis of the organs or tissues that show significantly different content provides a more effective method of selecting target genes, especially for elucidating secondary metabolism pathways. In addition, geniposide, which is an iridoid glucoside, is also a major active compound present in *G. jasminoides* fruits, and UGT proteins are also key enzymes in the biosynthetic pathway of geniposide. The amount of geniposide in green fruits was comparable to that in red fruits (Gao and Zhu, 2013). However, the crocin content increased during fruit development. Following the consistency of chemical contents and biosynthetic gene expression, the candidate UGTs whose expression in red fruits was 1.5 times higher than that in green fruits and whose FPKM value >100 may be involved in crocin biosynthesis. The UGT genes whose expression did not change significantly may be involved in geniposide biosynthesis.

In conclusion, the candidate genes that may encode key enzymes of the MEP, carotenoid and crocin biosynthesis pathways were identified by *de novo* transcriptome analysis of the green fruits, red fruits and leaves of *G. jasminoides*. In addition, the comprehensive and detailed relationships between candidate gene expression and crocin distribution were established. This study provides a molecular foundation for determining the crocin pathway. However, future functional characterizations of candidate genes *in vivo* and *in vitro* should be performed.

## Accession numbers

Transcriptome data from this study has been submitted to Sequence Read Archive (SRA) of the National Center for Biotechnology Information (NCBI) under the accession number PRJNA321361.

## Author contributions

JS, KH, and AJ designed this research. AJ and JJ performed the experiment, data analysis and wrote this paper. ZX and YL contributed to transcriptome data analysis and discussion. JS revised this paper. WB and CH carried out the chemicals detection. KH, FR, and JL collected the materials.

### Conflict of interest statement

The authors declare that the research was conducted in the absence of any commercial or financial relationships that could be construed as a potential conflict of interest.

## Supplementary material

Supplementary Figure 1 **Length distribution of the contigs, unigenes and CDSs**.

Supplementary Figure 2 **GO (A)** and KEGG **(B)** classifications of the unigenes.

Supplementary Figure 3 **Number of DEGs in comparisons of different *G. jasmonoides* organs**. Red bars represent the number of up-regulated genes in each group. Blue bars represent the number of down-regulated genes in each group. L, leaves; GF, green fruits; RF, red fruits.

Supplementary Figure 4 **Expression profile of the genes in the MEP pathway**.

Supplementary Figure 5 **Expression profile of the ALDH and UGT genes in *G. jasminoides***.

Supplementary Figure 6 **Validation of the expression of the genes related to crocin biosynthesis using qRT-PCR**. The characters on the x-axis indicate the leaves (L), green fruits (G), and red fruits (R). The y-axis represents the fold changes in gene expression. The *GAPDH* gene was used as an internal reference. The red star next to gene names represents the most likely genes involved in crocin biosynthesis. One-way ANOVA was performed using IBM SPSS 20 software. Asterisks represents significant differences from this comparison. *P* < 0.01 was considered highly significant.

Supplementary Figure 7 **Pigments produced in *E. coli* during functional analysis of CCD4a**. Decoloration of zeaxanthin was observed in CCD4a-expressing cells **(A)**. Results of HPLC analysis of zeaxanthin obtained from *E. coli* cells **(B)**. CCD4a, the pigments extracted from pET28a-CCD4a and pACCAR25ΔcrtX-expressing cells. CK, the pigments extracted from pACCAR25ΔcrtX-expressing cells.

Supplementary Figure 8 **Comparison of the CCDs (A)**, ALDHs **(B)**, and UGTs **(C)** proteins among different species. The 100% conservation of amino acid residues was indicated by gray background. The 75% conservation of amino acid residues was indicated by pink background. The 50% conservation of amino acid residues was indicated by blue background.

Supplementary Figure 9 **The distribution of the conserved motifs of CCDs (A)**, ALDHs **(B)**, and UGTs **(C)**.

Supplementary Table 1 **Primers for the qRT-PCR analysis**.

Supplementary Table 2 **Summary of the RNA-Seq data for each *G. jasmonoides* organ**.

Supplementary Table 3 **Annotation percentage of *G. jasminoides***.

Supplementary Table 4 **Overview of the differentially expressed genes of green fruits and leaves (A); red fruits and leaves (B); green fruits and red fruits (C)**.

Supplementary Table 5 **FPKM values of the pathway genes**.

Supplementary Table 6 **Summary of the 27 selected genes by DEGs analysis**.

Supplementary Table 7 **The physicochemical property of CCDs, ALDHs, and UGTs proteins, which used in phylogenetic analysis**.
