# Supplementary figures and images for: Transcriptome-Guided Mining of Genes Involved in Crocin Biosynthesis
Source: Front Plant Sci. 2017 Apr 11;8:518. doi: 10.3389/fpls.2017.00518 (PMC5387100; doi:10.3389/fpls.2017.00518)

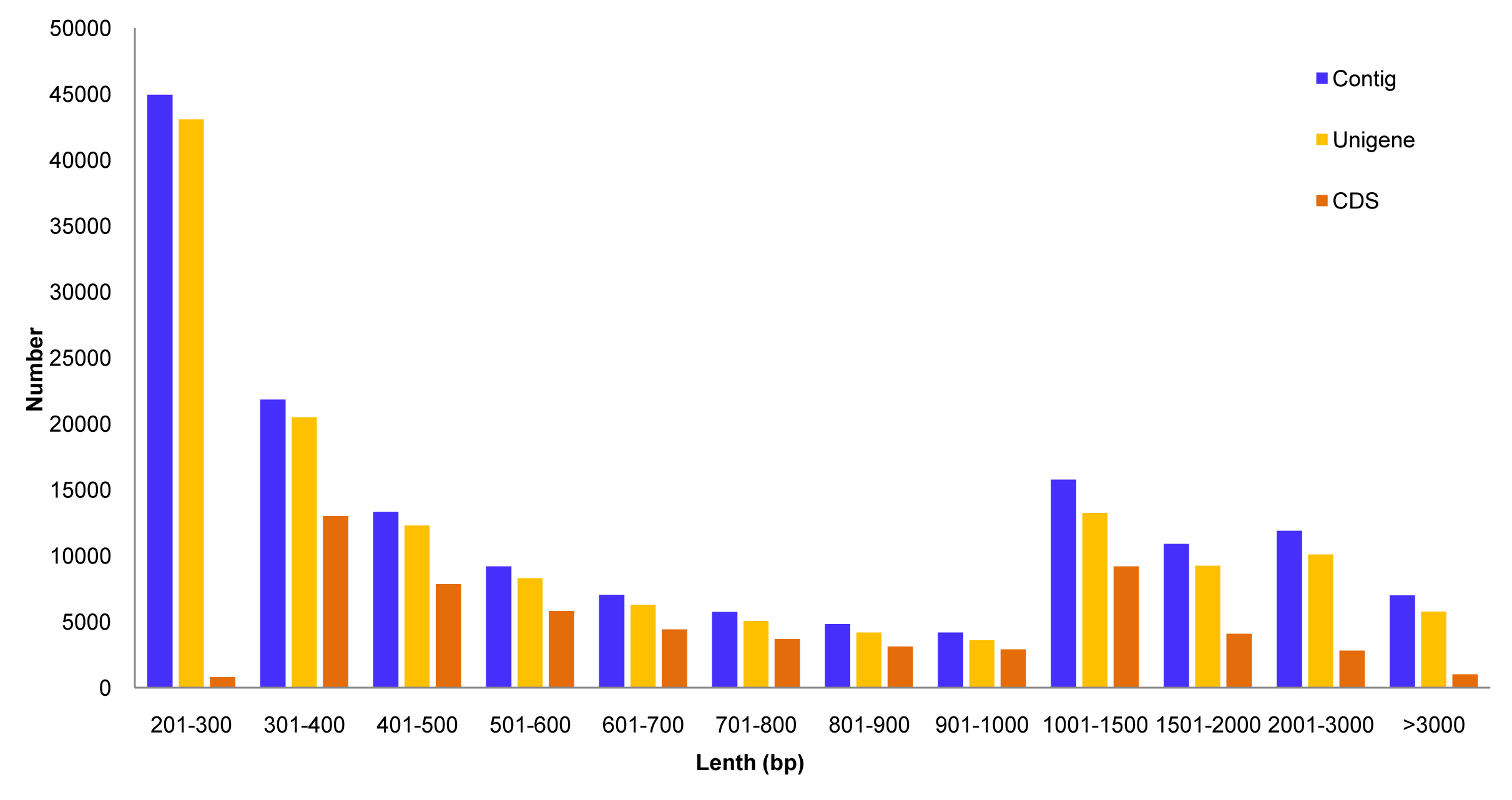

Supplement: Supplementary Figure 1 — Length distribution of the contigs, unigenes and CDSs. [file Image1.JPEG]

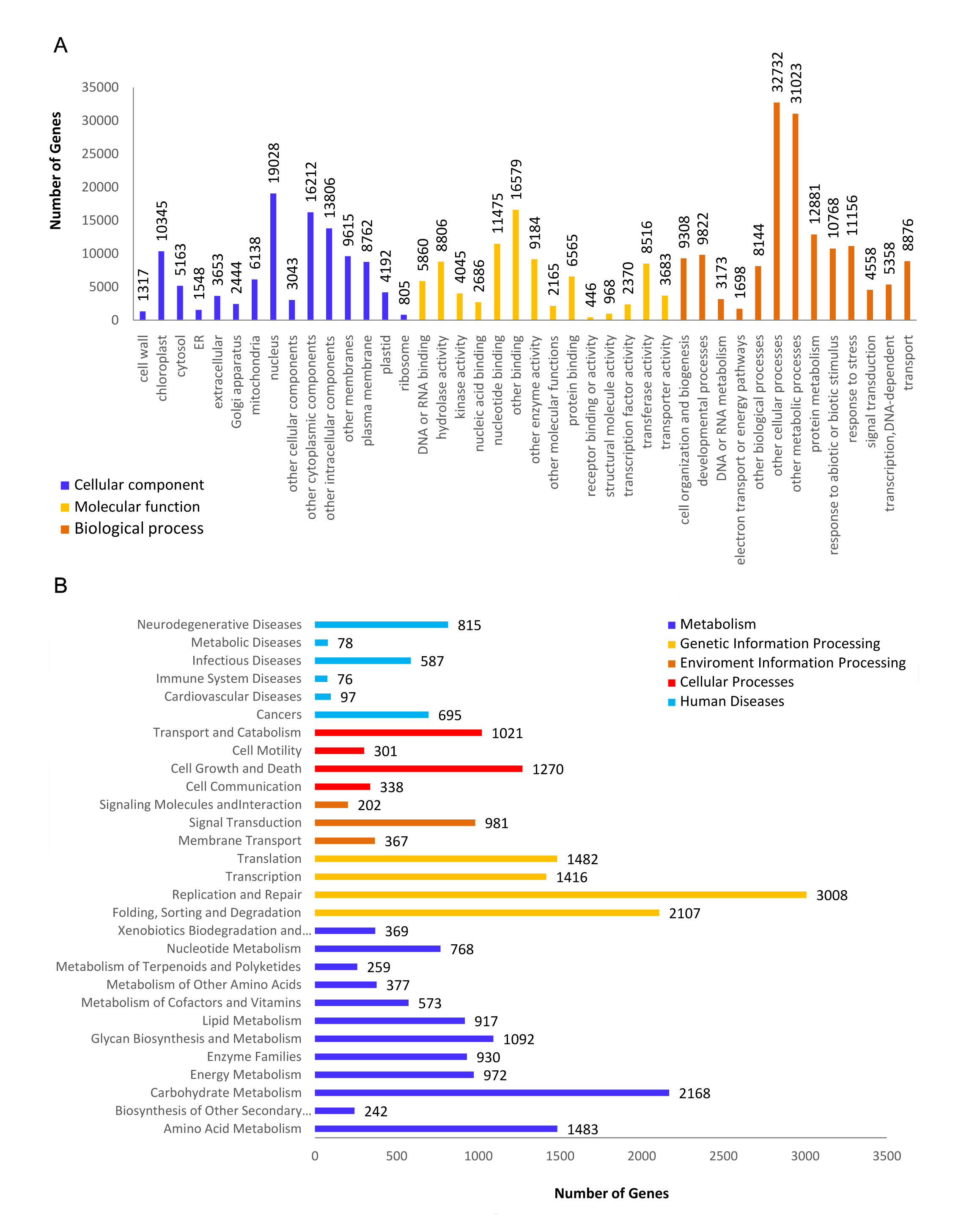

Supplement: Supplementary Figure 2 — GO (A) and KEGG (B) classifications of the unigenes. [file Image2.JPEG]

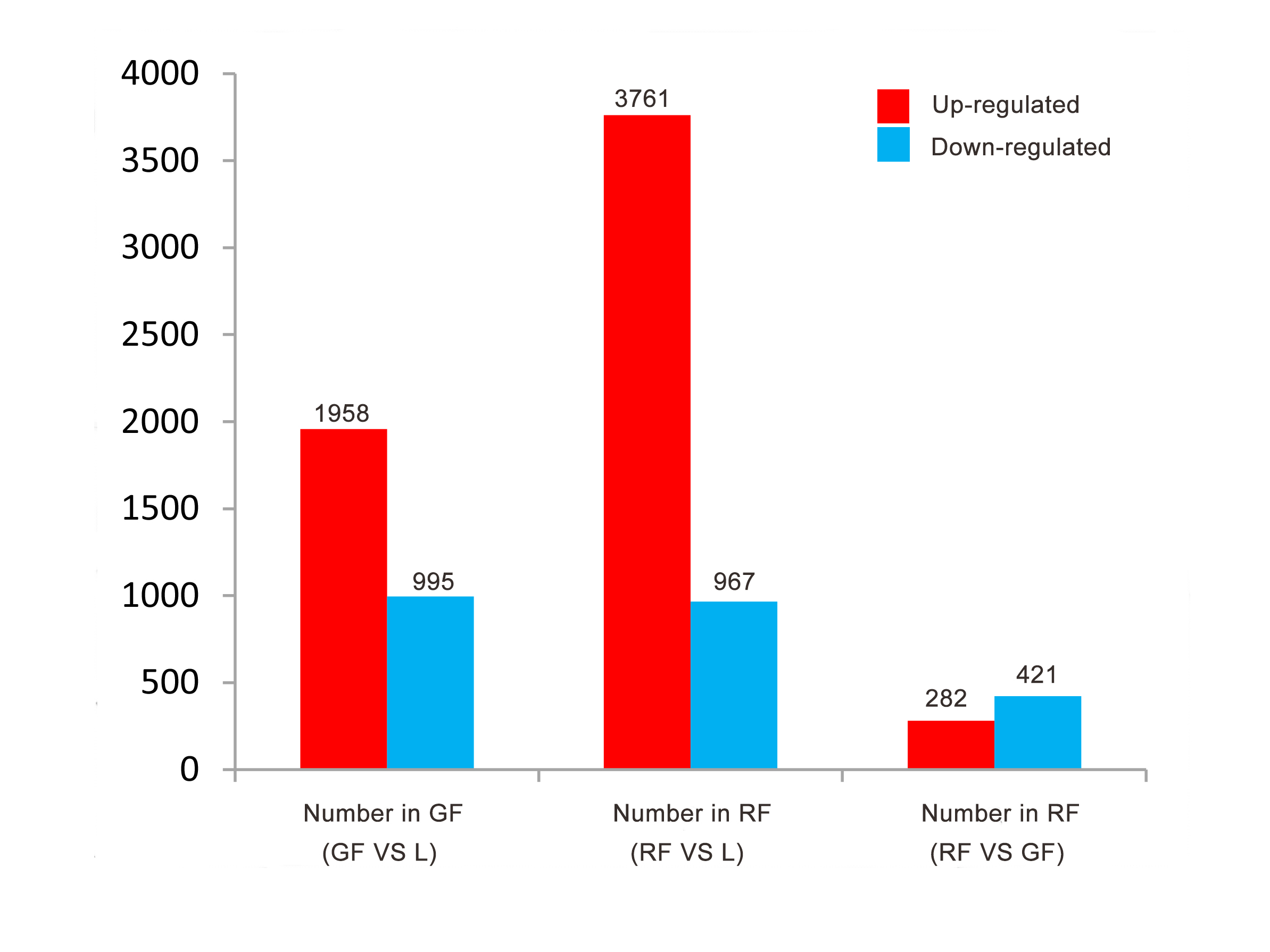

Supplement: Supplementary Figure 3 — Number of DEGs in comparisons of different G. jasmonoides organs. Red bars represent the number of up-regulated genes in each group. Blue bars represent the number of down-regulated genes in each group. L, leaves; GF, green fruits; RF, red fruits. [file Image3.JPEG]

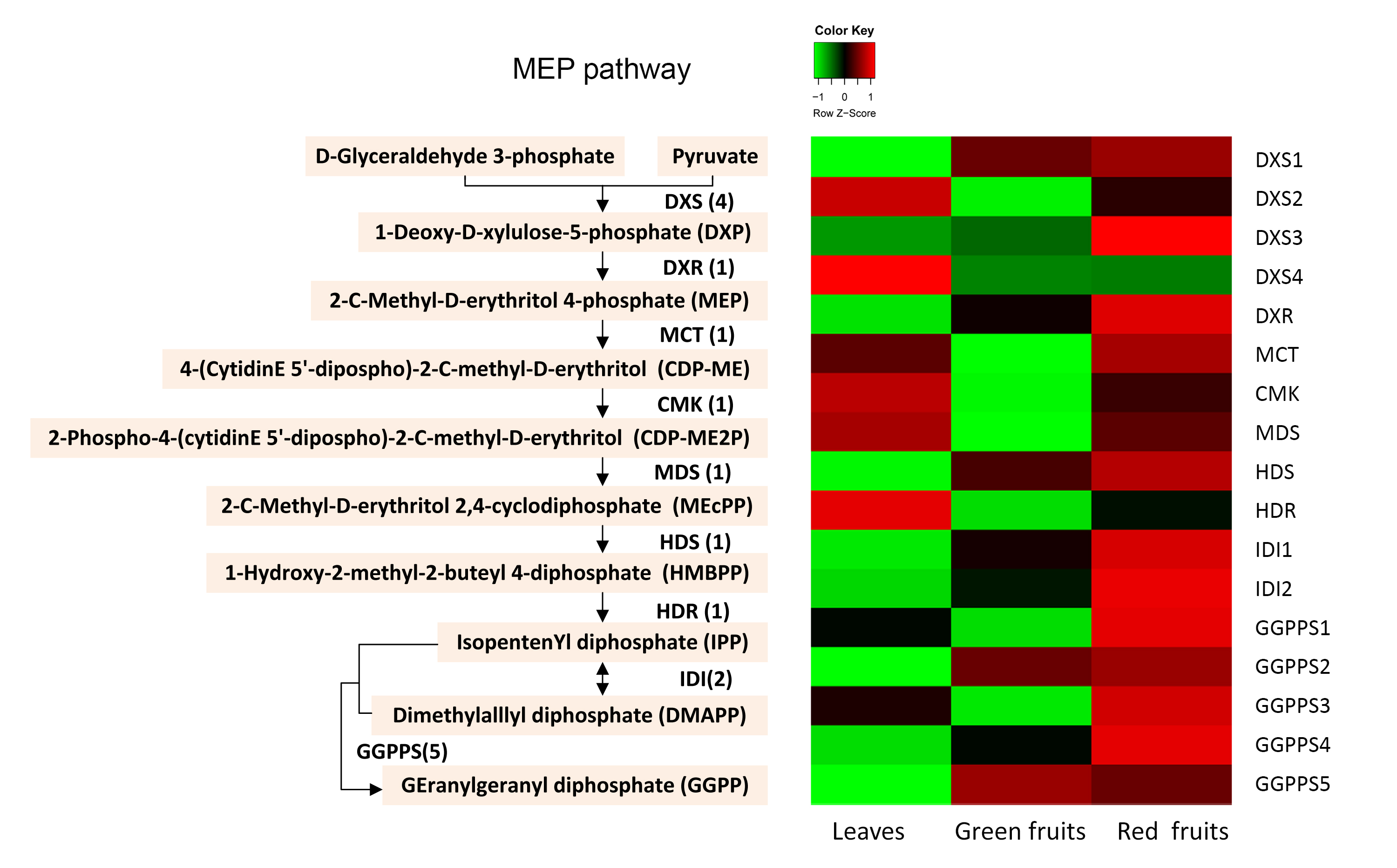

Supplement: Supplementary Figure 4 — Expression profile of the genes in the MEP pathway. [file Image4.JPEG]

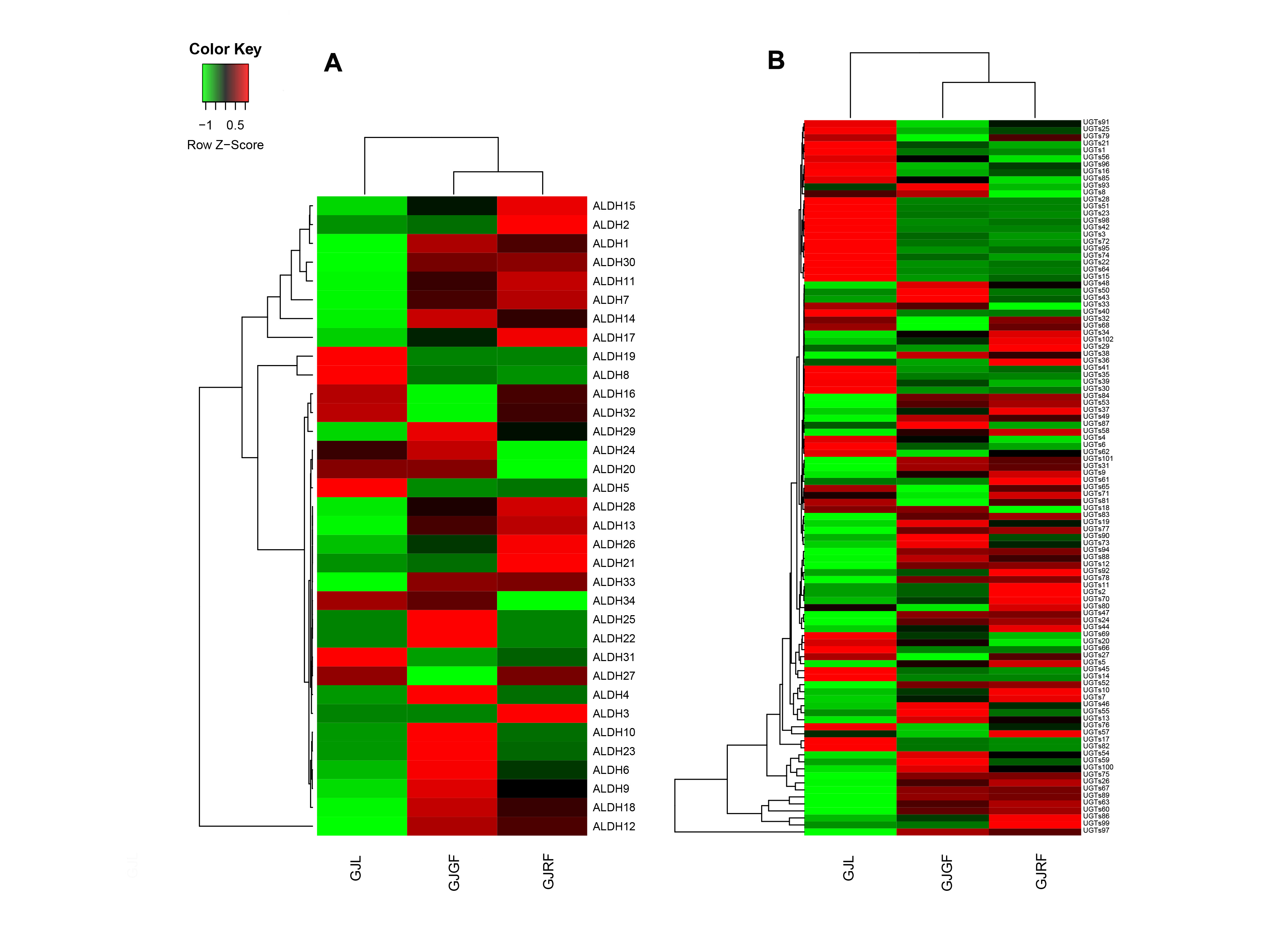

Supplement: Supplementary Figure 5 — Expression profile of the ALDH and UGT genes in G. jasminoides. [file Image5.JPEG]

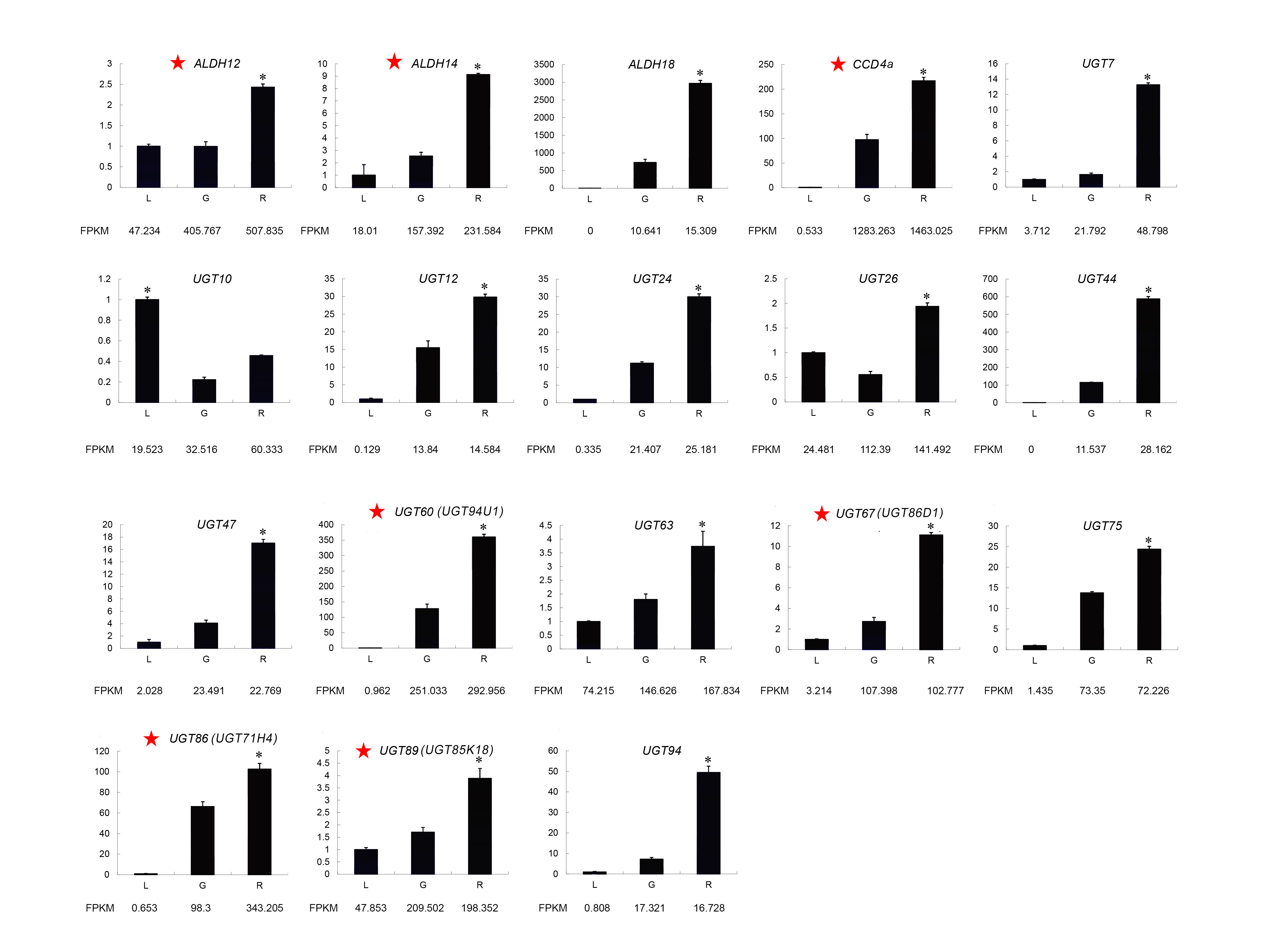

Supplement: Supplementary Figure 6 — Validation of the expression of the genes related to crocin biosynthesis using qRT-PCR. The characters on the x-axis indicate the leaves (L), green fruits (G), and red fruits (R). The y-axis represents the fold changes in gene expression. The GAPDH gene was used as an internal reference. The red star next to gene names represents the most likely genes involved in crocin biosynthesis. One-way ANOVA was performed using IBM SPSS 20 software. Asterisks represents significant differences from this comparison. P < 0.01 was considered highly significant. [file Image6.JPEG]

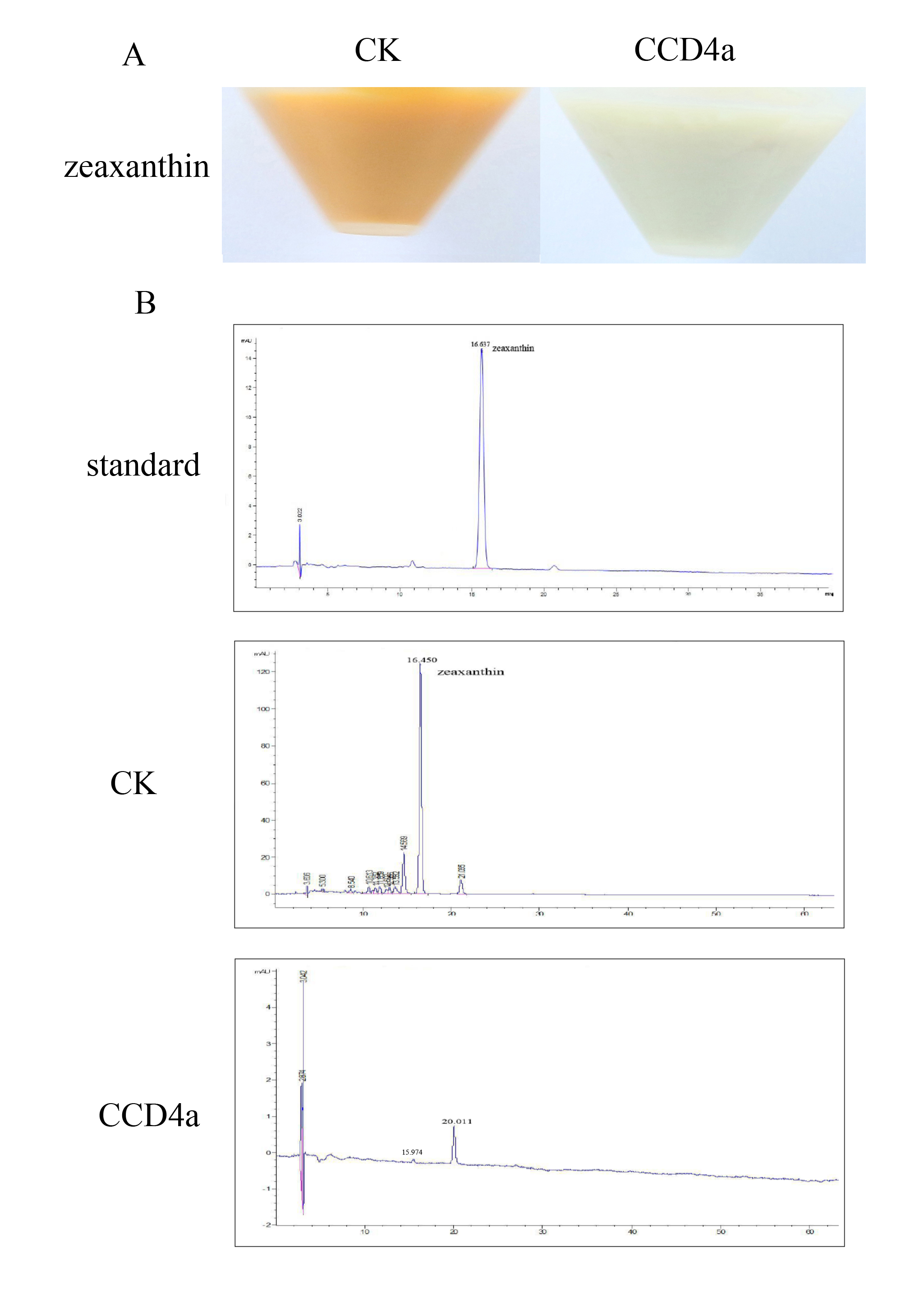

Supplement: Supplementary Figure 7 — Pigments produced in E. coli during functional analysis of CCD4a. Decoloration of zeaxanthin was observed in CCD4a-expressing cells (A). Results of HPLC analysis of zeaxanthin obtained from E. coli cells (B). CCD4a, the pigments extracted from pET28a-CCD4a and pACCAR25ΔcrtX-expressing cells. CK, the pigments extracted from pACCAR25ΔcrtX-expressing cells. [file Image7.JPEG]

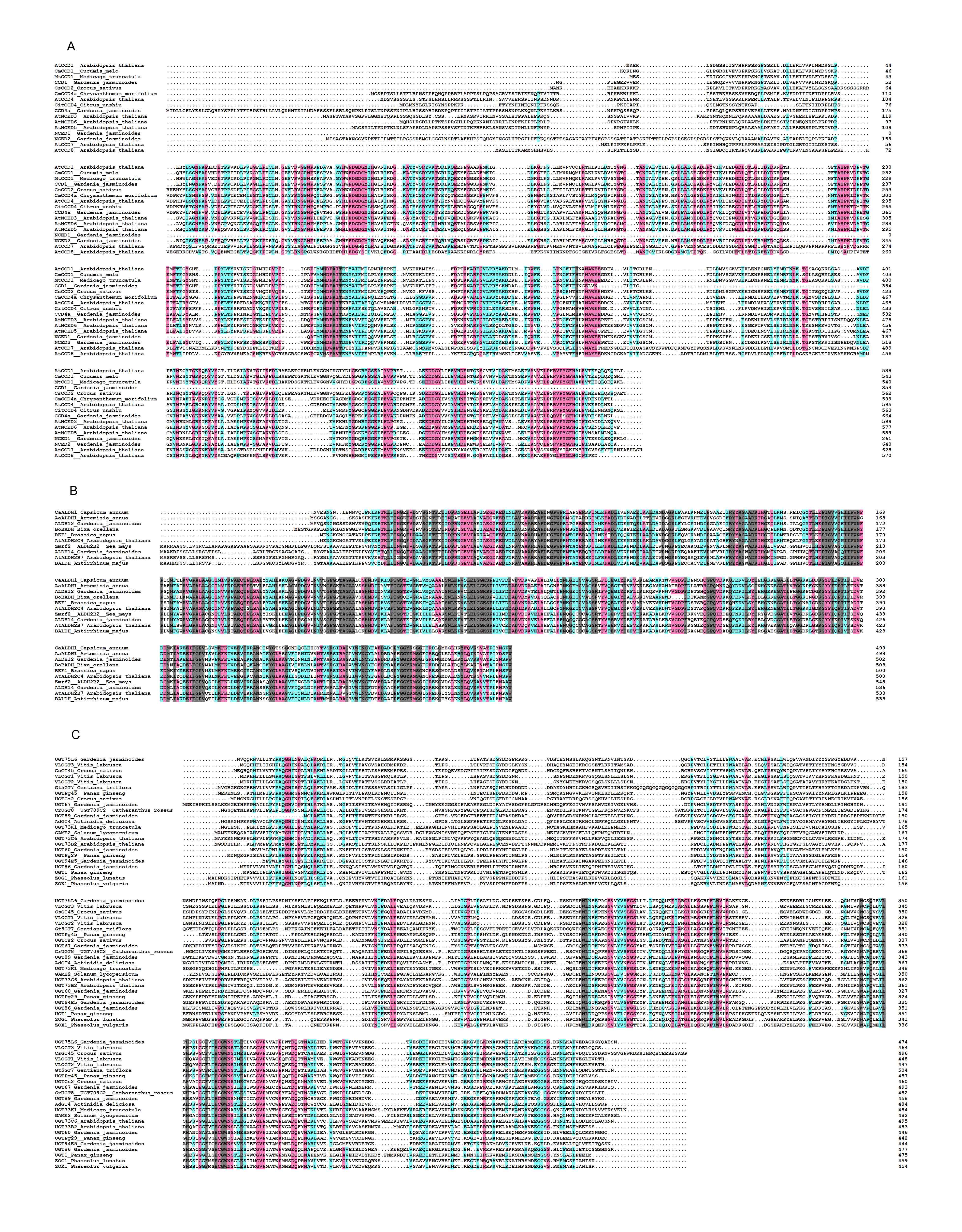

Supplement: Supplementary Figure 8 — Comparison of the CCDs (A), ALDHs (B), and UGTs (C) proteins among different species. The 100% conservation of amino acid residues was indicated by gray background. The 75% conservation of amino acid residues was indicated by pink background. The 50% conservation of amino acid residues was indicated by blue background. [file Image8.JPEG]

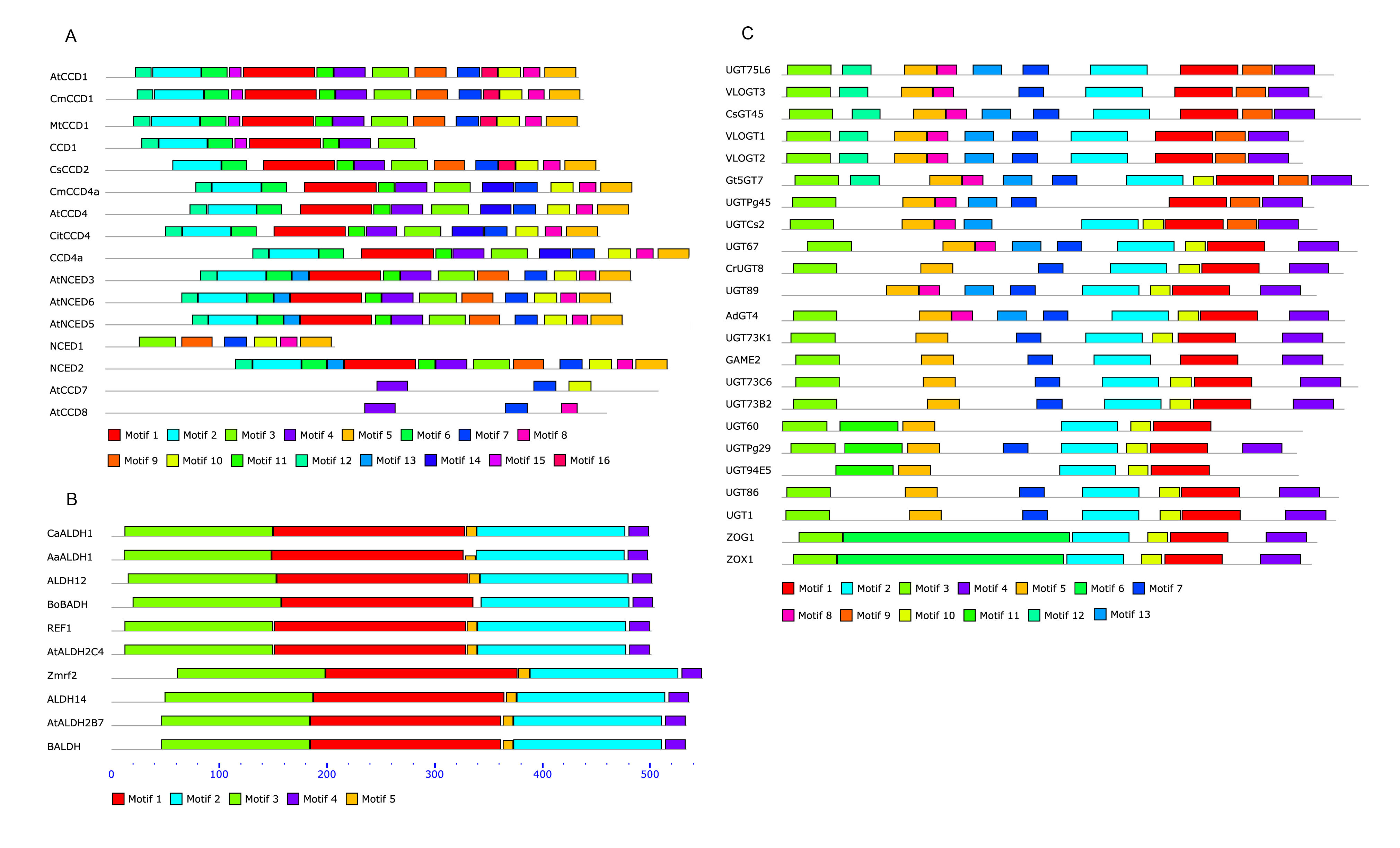

Supplement: Supplementary Figure 9 — The distribution of the conserved motifs of CCDs (A), ALDHs (B), and UGTs (C). [file Image9.JPEG]
