# Supplementary material for: Transcriptome-Guided Mining of Genes Involved in Crocin Biosynthesis
Source: Front Plant Sci. 2017 Apr 11;8:518. doi: 10.3389/fpls.2017.00518 (PMC5387100; doi:10.3389/fpls.2017.00518)
Supplement: Supplementary Table 1 — Primers for the qRT-PCR analysis. [file Table1.DOCX]

**Supplemental Table 1. Primers for qRT-PCR in this study**

| **Primer** | **Sequence (5’ to 3’)** |
| --- | --- |
| **ALDH12 qF1** | TCACGGATGAGATGACTATTGC |
| **ALDH12 qR1** | GTGTTGGCGATGTTAATGTTGT |
| **ALDH14qF1** | GCCATTCTTCTCCTAGCCTTC |
| **ALDH14 qR1** | GATACCGCCACCTTGATACG |
| **ALDH18 qF1** | CGATTGAACTTGGTGTTGTGAA |
| **ALDH18 qR1** | GCTACAGCCTCATCGTAATTCT |
| **CCD4a qF1** | CCTTCCACCACCTCACATTC |
| **CCD4a qR1** | AGCCAGTAGAACCATCATTGC |
| **UGTs7 qF1** | GCCAACTGCCATAACTGACA |
| **UGTs7 qR1** | AACTCCTGACTTGCCATAAGC |
| **UTGS10 QF1** | CAGGCAGATTATGTCCTTGTCA |
| **UTGS10 QR1** | GTCGGCTGAAGCAATTCCA |
| **UTGS12 qF1** | ACTCAGAACAACAGTTGAATGC |
| **UTGS12 qR1** | TCCTCACTTCGCTATCACTCT |
| **UTGS24 qF1** | CTCTTCTACTGCGGATTGCTTA |
| **UTGS24 qR1** | TTGTACCGTCTTGGCTGTTC |
| **UTGS26 qF1** | GAGACGAACTTGTGACATCCA |
| **UTGS26 qR1** | CGACTAACACCACCTTCCATAA |
| **UGTS44 qF1** | GGTATAGTGAATAGCGGCGTAA |
| **UGTS44 QR1** | CCATCCTACCATACAGCCTCT |
| **UGTS47 QF1** | CTAAGCCGAGGACACTATTACC |
| **UGTS47 QR1** | CAAGTCACCAAGGCAGAACA |
| **UGTS54 QF1** | ACAGAATGCGATGAAGTGGAA |
| **UGTS54 QR1** | ACAGAGCTACCGTCAGTGG |
| **UTGS60 qF1** | CCTCATTACCACACCACCAAT |
| **UTGS60 qR1** | AGTCGTACACAACCAAGTCAG |
| **UTGS63 qF1** | GAAGACAAGAATCCAGTCACCT |
| **UTGS63 qR1** | ACCGTGCCAGAACTCCAA |
| **UTGS67 qF1** | ATTCTCAGCAACCTCTTCGC |
| **UTGS67 qR1** | AACACATTGTGGATGGAACTCT |
| **UGTS75 QF1** | AATGGAGTAGTTGTGCCTTGG |
| **UGTS75 QR1** | TATCTGCTTGCTGTTCGTGTAT |
| **UTGS86 qF1** | GCCACATCAAGTCGGTCTG |
| **UTGS86 qR1** | GGCTTCTTGTCTTCACTTCCT |
| **UGTS89 QF1** | GCGAGGAAGCACAGAGTTG |
| **UGTS89 QR1** | GACTTGGGTTTCAGGAACAATG |
| **UGTS94 QF1** | GCAGAGCTAGAACATGAACCT |
| **UGTS94 QR1** | CCAACCAGCAAGCCAGTAG |
| **GAPDH QF1** | TCTACTGTTGGAACTCGGAATG |
| **GAPDH QR1** | CGCAGAAGACTGTTGATGGT |
| **ACTIN QF1** | TCCTCTTCCAGCCTTCTATCAT |
| **ACTIN QR1** | GAACCACCACTGAGCACAAT |
