# Supplementary material for: Transcriptome-Guided Mining of Genes Involved in Crocin Biosynthesis
Source: Front Plant Sci. 2017 Apr 11;8:518. doi: 10.3389/fpls.2017.00518 (PMC5387100; doi:10.3389/fpls.2017.00518)
Supplement: Supplementary Table 2 — Summary of the RNA-Seq data for each G. jasmonoides organ. [file Table2.DOCX]

**SupplementalTable 2. Summary of** **RNA-Seq data for each sample**

| **Item** | **Raw reads number** | **Clean reads number** | **Clean bases** | **Average length (bp)** | **Q20 (%)** | **GC (%)** |
| --- | --- | --- | --- | --- | --- | --- |
| **Leaves** | 45,224,600 | 43,669,608 | 4,272,485,445 | 97.84 | 99.61 | 43.85 |
| **Green fruits** | 46,735,458 | 45,146,608 | 4,410,678,321 | 97.70 | 99.60 | 44.80 |
| **Red fruits** | 60,458,180 | 58,351,114 | 5,687,310,533 | 97.47 | 99.57 | 46.01 |
