# Supplementary material for: Transcriptome-Guided Mining of Genes Involved in Crocin Biosynthesis
Source: Front Plant Sci. 2017 Apr 11;8:518. doi: 10.3389/fpls.2017.00518 (PMC5387100; doi:10.3389/fpls.2017.00518)
Supplement: Supplementary Table 3 — Annotation percentage of G. jasminoides. [file Table3.DOCX]

**Supplemental Table 3. The annotation percentage of *G. jasminoides***

| **Database** | **Number of unigenes** | **Annotation percentage (%)** |
| --- | --- | --- |
| **Uniprot** | 37,961 | 64.67 |
| **Pfam** | 41,907 | 71.39 |
| **KEGG** | 11,375 | 19.38 |
| **GO** | 45,715 | 77.88 |
| **annotated CDS** | 52,816 | 89.97 |
| **Total CDS** | 58,702 | 100 |
| **Total unigenes** | 141,665 |  |
