# Supplementary material for: Transcriptome-Guided Mining of Genes Involved in Crocin Biosynthesis
Source: Front Plant Sci. 2017 Apr 11;8:518. doi: 10.3389/fpls.2017.00518 (PMC5387100; doi:10.3389/fpls.2017.00518)
Supplement: Supplementary Table 5 — FPKM values of the pathway genes. [file Table5.DOCX]

**Supplemental Table 5. FPKM value of the pathway genes**

| **name** | **gene ID number** | **GJRF_RSEM** | **GJGF_RSEM** | **GJL_RSEM** |
| --- | --- | --- | --- | --- |
| ***DXS1*** | c100094_g2_i1\|m.51756 | 198.799 | 174.703 | 2.097 |
| ***DXS2*** | c102132_g4_i5\|m.65144 | 16.448 | 14.612 | 17.503 |
| ***DXS3*** | c88994_g1_i1\|m.17964 | 1.809 | 1.063 | 0.962 |
| ***DXS4*** | c98641_g3_i2\|m.43881 | 3.484 | 3.366 | 13.198 |
| ***DXR*** | c83277_g1_i2\|m.12784 | 103.291 | 83.699 | 61.086 |
| ***MCT*** | c97006_g1_i1\|m.36562 | 23.562 | 17.957 | 22.513 |
| ***CMK*** | c92044_g1_i2\|m.22893 | 36.046 | 19.645 | 43.058 |
| ***MDS*** | c91928_g1_i1\|m.22651 | 71.634 | 41.625 | 78.684 |
| ***HDS*** | c95826_g1_i1\|m.32458 | 150.816 | 144.865 | 128.049 |
| ***HDR*** | c97184_g1_i1\|m.37303 | 195.382 | 166.511 | 229.254 |
| ***IDI1*** | c96616_g2_i1\|m.35100 | 70.171 | 44.47 | 9.443 |
| ***IDI2*** | c12016_g1_i1\|m.752 | 1.452 | 0.625 | 0 |
| ***AACT1*** | c100033_g4_i9\|m.51264 | 67.089 | 57.05 | 47.518 |
| ***AACT2*** | c132686_g1_i1\|m.80742 | 1.117 | 0 | 0 |
| ***AACT3*** | c63592_g1_i1\|m.5068 | 1.932 | 0.261 | 0 |
| ***AACT4*** | c81976_g1_i1\|m.11933 | 332.932 | 306.332 | 128.014 |
| ***AACT5*** | c89914_g2_i1\|m.19223 | 75.62 | 78.884 | 25.228 |
| ***AACT6*** | c91367_g1_i1\|m.21620 | 3.495 | 1.626 | 0 |
| ***AACT7*** | c92857_g1_i1\|m.24719 | 15.89 | 10.015 | 0 |
| ***AACT8*** | c95012_g1_i1\|m.30087 | 206.102 | 186.771 | 85.137 |
| ***AACT9*** | c95308_g1_i1\|m.30984 | 22.691 | 5.419 | 0.034 |
| ***HMGS1*** | c17196_g1_i1\|m.1323 | 2.468 | 0.5 | 0 |
| ***HMGS2*** | c35320_g1_i1\|m.2833 | 1.306 | 0.834 | 0 |
| ***HMGS3*** | c86114_g1_i1\|m.14982 | 74.381 | 78.279 | 42.457 |
| ***HMGS4*** | c79356_g1_i1\|m.10644 | 2.512 | 0.823 | 0 |
| ***HMGR1*** | c102148_g2_i1\|m.65227 | 25.404 | 36.873 | 8.378 |
| ***HMGR2*** | c1805_g1_i1\|m.225 | 2.155 | 0.063 | 0 |
| ***HMGR3*** | c73469_g2_i1\|m.7932 | 0.335 | 0.636 | 0 |
| ***HMGR4*** | c97937_g3_i1\|m.40618 | 17.67 | 15.19 | 4.91 |
| ***MK1*** | c131738_g1_i1\|m.80530 | 1.217 | 0.386 | 0 |
| ***MK2*** | c69092_g1_i1\|m.6541 | 25.069 | 27.816 | 9.486 |
| ***MK3*** | c85771_g1_i1\|m.14583 | 1.329 | 0.125 | 0 |
| ***PMK*** | c103193_g1_i1\|m.72899 | 7.348 | 10.412 | 10.698 |
| ***MDC1*** | c83206_g2_i1\|m.12766 | 1.541 | 1.292 | 0 |
| ***MDC2*** | c95489_g1_i1\|m.31497 | 34.862 | 26.451 | 6.72 |
| ***MDC3*** | c52077_g1_i1\|m.4133 | 1.016 | 0.188 | 0 |
| ***GPPS1*** | c96816_g1_i1\|m.35844 | 51.255 | 43.939 | 166.183 |
| ***GPPS2*** | c98338_g3_i1\|m.42504 | 20.055 | 17.217 | 22.255 |
| ***GGPPS1*** | c90588_g1_i1\|m.20239 | 34.17 | 22.907 | 28.399 |
| ***GGPPS2*** | c86896_g1_i1\|m.15808 | 268.501 | 238.631 | 20.038 |
| ***GGPPS3*** | c66399_g2_i1\|m.5774 | 2.512 | 0.448 | 1.676 |
| ***GGPPS4*** | c90457_g2_i1\|m.20078 | 367.18 | 203.968 | 49.056 |
| ***GGPPS5*** | c90484_g1_i1\|m.20095 | 4.612 | 5.013 | 1.719 |
| ***PSY1*** | c91475_g1_i3\|m.21840 | 795.642 | 630.152 | 201.62 |
| ***PSY2*** | c94591_g1_i3\|m.28940 | 4.165 | 3.012 | 26.036 |
| ***PDS1*** | c98273_g1_i12\|m.42160 | 97.272 | 107.294 | 107.332 |
| ***PDS2*** | c89992_g1_i1\|m.19381 | 399.373 | 318.004 | 61.85 |
| ***ZISO*** | c95374_g1_i1\|m.31151 | 51.165 | 70.358 | 13.473 |
| ***ZDS*** | c98273_g1_i12\|m.42160 | 97.272 | 107.294 | 107.332 |
| ***CRTISO1*** | c100405_g1_i3\|m.53542 | 29.078 | 33.017 | 28.055 |
| ***CRTISO2*** | c101479_g3_i1\|m.60418 | 11.58 | 11.547 | 63.827 |
| ***LCYB1*** | c67606_g1_i1\|m.6029 | 13.277 | 13.569 | 27.703 |
| ***LCYB2*** | c93647_g1_i1\|m.26450 | 81.84 | 55.455 | 1.229 |
| ***LCYB3*** | c94726_g3_i1\|m.29241 | 3.707 | 7.545 | 26.766 |
| ***CHY1*** | c99414_g2_i1\|m.47641 | 148.817 | 189.835 | 103.792 |
| ***CYP97A51*** | c102309_g2_i1\|m.66352 | 146.34 | 81.29 | 10.26 |
| ***CCD1*** | c102279_g2_i2\|m.66102 | 176.767 | 185.583 | 177.93 |
| ***CCD4a*** | c97922_g1_i2\|m.40550 | 1463.025 | 1283.263 | 0.533 |
| ***CCD4b*** | c88487_g3_i1\|m.17423 | 6.376 | 15.664 | 64.557 |
| ***NCED1*** | c86119_g2_i1\|m.14984 | 0.514 | 2.011 | 0 |
| ***NCED2*** | c97123_g1_i1\|m.37001 | 4.355 | 13.726 | 4.606 |
| ***ALDH1*** | c83315_g1_i2\|m.12806 | 85.246 | 79.655 | 60.785 |
| ***ALDH2*** | c100696_g4_i13\|m.55357 | 69.735 | 81.187 | 68.622 |
| ***ALDH3*** | c42940_g1_i1\|m.3343 | 0 | 5.378 | 0 |
| ***ALDH4*** | c87352_g1_i1\|m.16132 | 3.752 | 0.396 | 0 |
| ***ALDH5*** | c98848_g1_i2\|m.44868 | 0.223 | 0.74 | 10.114 |
| ***ALDH6*** | c95617_g1_i1\|m.31837 | 7.861 | 2.355 | 0 |
| ***ALDH7*** | c93337_g1_i1\|m.25772 | 125.111 | 136.215 | 92.518 |
| ***ALDH8*** | c90552_g1_i1\|m.20173 | 63.538 | 52.381 | 214.526 |
| ***ALDH9*** | c92803_g1_i1\|m.24604 | 6.767 | 3.377 | 0 |
| ***ALDH10*** | c97281_g1_i1\|m.37639 | 11.133 | 1.094 | 0 |
| ***ALDH11*** | c85778_g1_i1\|m.14595 | 60.668 | 88.92 | 0.369 |
| ***ALDH12*** | c7348_g1_i1\|m.555 | 507.835 | 405.767 | 47.234 |
| ***ALDH13*** | c73873_g1_i1\|m.8065 | 0.648 | 0.886 | 0 |
| ***ALDH14*** | c91898_g1_i1\|m.22588 | 231.584 | 157.392 | 18.01 |
| ***ALDH15*** | c97778_g1_i1\|m.39935 | 79.964 | 92.203 | 70.684 |
| ***ALDH16*** | c89561_g1_i9\|m.18705 | 19.776 | 20.813 | 21.172 |
| ***ALDH17*** | c98450_g9_i1\|m.43060 | 127.735 | 324.31 | 0.318 |
| ***ALDH18*** | c94315_g1_i1\|m.28252 | 15.309 | 10.641 | 0 |
| ***ALDH19*** | c94548_g1_i1\|m.28825 | 62.03 | 61.948 | 303.108 |
| ***ALDH20*** | c101391_g1_i6\|m.59840 | 6.767 | 5.982 | 6.771 |
| ***ALDH21*** | c83008_g1_i1\|m.12667 | 0.134 | 1.146 | 0.043 |
| ***ALDH22*** | c78033_g1_i1\|m.9962 | 0.592 | 0 | 0 |
| ***ALDH23*** | c90219_g1_i1\|m.19726 | 9.335 | 1.115 | 0 |
| ***ALDH24*** | c98716_g1_i3\|m.44187 | 7.738 | 5.326 | 6.994 |
| ***ALDH25*** | c83536_g1_i1\|m.12911 | 0.994 | 0 | 0 |
| ***ALDH26*** | c104532_g1_i1\|m.75509 | 0.38 | 0.74 | 0.215 |
| ***ALDH27*** | c121710_g1_i1\|m.78744 | 0 | 0.834 | 0.902 |
| ***ALDH28*** | c50242_g1_i1\|m.3958 | 0.625 | 1.063 | 0 |
| ***ALDH29*** | c90945_g1_i5\|m.20885 | 36.157 | 25.367 | 16.627 |
| ***ALDH30*** | c97632_g1_i1\|m.39279 | 78.177 | 80.416 | 29.937 |
| ***ALDH31*** | c76191_g1_i1\|m.9019 | 0 | 0.24 | 1.667 |
| ***ALDH32*** | c100642_g1_i1\|m.55005 | 11.222 | 16.164 | 18.217 |
| ***ALDH33*** | c110451_g1_i1\|m.76611 | 1.273 | 1.23 | 0 |
| ***ALDH34*** | c16504_g1_i1\|m.1218 | 0.826 | 0 | 0.971 |
| ***UGT1*** | c102314_g1_i1\|m.66408 | 5.751 | 6.409 | 16.807 |
| ***UGT2*** | c101535_g1_i1\|m.60824 | 16.426 | 3.074 | 0.498 |
| ***UGT3*** | c100121_g1_i2\|m.51875 | 2.077 | 2.949 | 9.031 |
| ***UGT4*** | c100320_g2_i2\|m.53092 | 0.849 | 2.762 | 4.881 |
| ***UGT5*** | c100412_g1_i1\|m.53615 | 30.328 | 25.961 | 19.626 |
| ***UGT6*** | c100615_g1_i1\|m.54886 | 0.86 | 1.376 | 3.532 |
| ***UGT7*** | c100985_g2_i1\|m.57145 | 48.798 | 21.792 | 3.712 |
| ***UGT8*** | c101041_g1_i3\|m.57499 | 6.644 | 15.935 | 13.671 |
| ***UGT9*** | c101144_g1_i1\|m.58185 | 8.408 | 5.742 | 1.959 |
| ***UGT10*** | c101186_g1_i1\|m.58441 | 60.333 | 32.516 | 19.523 |
| ***UGT11*** | c101535_g1_i2\|m.60825 | 16.426 | 3.074 | 0.498 |
| ***UGT12*** | c101562_g1_i1\|m.60948 | 14.584 | 13.84 | 0.129 |
| ***UGT13*** | c101757_g3_i1\|m.62394 | 33.008 | 50.963 | 10.689 |
| ***UGT14*** | c101769_g1_i1\|m.62554 | 1.686 | 1.626 | 38.263 |
| ***UGT15*** | c101874_g3_i1\|m.63322 | 6.599 | 4.429 | 24.618 |
| ***UGT16*** | c101890_g1_i2\|m.63432 | 7.459 | 6.993 | 9.254 |
| ***UGT17*** | c101925_g2_i2\|m.63655 | 20.837 | 25.523 | 109.901 |
| ***UGT18*** | c101981_g1_i1\|m.64057 | 2.591 | 7.514 | 7.476 |
| ***UGT19*** | c102020_g1_i1\|m.64350 | 6.934 | 8.4 | 5.774 |
| ***UGT20*** | c102161_g1_i10\|m.65383 | 10.653 | 23.543 | 32.987 |
| ***UGT21*** | c102326_g1_i6\|m.66485 | 4.735 | 7.806 | 18.466 |
| ***UGT22*** | c102458_g1_i1\|m.67454 | 5.226 | 3.981 | 20.786 |
| ***UGT23*** | c102583_g1_i1\|m.68434 | 0.368 | 0.959 | 13.602 |
| ***UGT24*** | c102815_g1_i1\|m.70065 | 25.181 | 21.407 | 0.335 |
| ***UGT25*** | c102870_g1_i1\|m.70473 | 11.412 | 9.505 | 17.074 |
| ***UGT26*** | c102877_g1_i4\|m.70555 | 141.492 | 112.39 | 24.481 |
| ***UGT27*** | c103009_g1_i1\|m.71612 | 34.963 | 23.501 | 37.825 |
| ***UGT28*** | c103286_g1_i1\|m.73551 | 0.581 | 0.657 | 12.313 |
| ***UGT29*** | c104007_g1_i1\|m.75371 | 0.681 | 0.271 | 0.318 |
| ***UGT30*** | c109205_g1_i1\|m.76281 | 0.078 | 0 | 1.212 |
| ***UGT31*** | c130669_g1_i1\|m.80248 | 5.282 | 5.722 | 2.896 |
| ***UGT32*** | c65873_g1_i1\|m.5664 | 1.05 | 0.24 | 1.04 |
| ***UGT33*** | c66178_g2_i1\|m.5728 | 0.246 | 1.23 | 1.469 |
| ***UGT34*** | c69475_g1_i1\|m.6675 | 0.96 | 0.5 | 0 |
| ***UGT35*** | c74536_g2_i1\|m.8361 | 0.145 | 0.188 | 2.157 |
| ***UGT36*** | c79292_g1_i1\|m.10620 | 2.524 | 0 | 0.438 |
| ***UGT37*** | c81167_g1_i1\|m.11501 | 2.635 | 1.115 | 0.189 |
| ***UGT38*** | c81642_g2_i1\|m.11741 | 0.782 | 1.157 | 0 |
| ***UGT39*** | c82792_g1_i1\|m.12480 | 0.223 | 0.865 | 2.741 |
| ***UGT40*** | c82932_g1_i1\|m.12616 | 0.569 | 0.552 | 1.495 |
| ***UGT41*** | c83090_g3_i1\|m.12722 | 0.458 | 0.188 | 2.629 |
| ***UGT42*** | c83856_g1_i1\|m.13061 | 0 | 0 | 10.346 |
| ***UGT43*** | c84743_g1_i1\|m.13718 | 0.927 | 1.761 | 0.773 |
| ***UGT44*** | c85148_g1_i1\|m.14007 | 28.162 | 11.537 | 0 |
| ***UGT45*** | c86012_g1_i1\|m.14844 | 16.906 | 20.49 | 59.78 |
| ***UGT46*** | c86738_g1_i1\|m.15650 | 19.274 | 39.708 | 8.077 |
| ***UGT47*** | c87823_g1_i1\|m.16650 | 22.769 | 23.491 | 2.028 |
| ***UGT48*** | c87993_g1_i1\|m.16869 | 0.96 | 1.063 | 0.842 |
| ***UGT49*** | c88169_g1_i1\|m.17090 | 2.144 | 2.803 | 0.146 |
| ***UGT50*** | c88323_g1_i2\|m.17237 | 0.804 | 1.042 | 0.791 |
| ***UGT51*** | c88446_g2_i2\|m.17368 | 0.648 | 0.823 | 11.085 |
| ***UGT52*** | c90097_g1_i1\|m.19503 | 42.779 | 41.886 | 28.811 |
| ***UGT53*** | c91003_g2_i2\|m.20970 | 1.854 | 1.522 | 0.172 |
| ***UGT54*** | c91161_g2_i1\|m.21232 | 82.666 | 125.553 | 38.195 |
| ***UGT55*** | c91209_g1_i1\|m.21344 | 8.386 | 27.92 | 6.427 |
| ***UGT56*** | c91249_g1_i1\|m.21407 | 5.315 | 9.974 | 14.53 |
| ***UGT57*** | c91379_g1_i1\|m.21643 | 98.746 | 13.674 | 43.703 |
| ***UGT58*** | c91838_g1_i2\|m.22487 | 2.914 | 2.48 | 1.684 |
| ***UGT59*** | c91876_g1_i1\|m.22531 | 55.945 | 126.616 | 41.236 |
| ***UGT60(UGT94U1)*** | c91895_g1_i1\|m.22581 | 292.956 | 251.033 | 0.962 |
| ***UGT61*** | c91986_g1_i1\|m.22780 | 6.242 | 0.625 | 0.928 |
| ***UGT62*** | c92613_g2_i1\|m.24217 | 2.881 | 1.751 | 4.021 |
| ***UGT63*** | c92767_g1_i1\|m.24506 | 167.834 | 146.626 | 74.215 |
| ***UGT64*** | c92903_g1_i2\|m.24833 | 2.892 | 2.168 | 21.516 |
| ***UGT65*** | c93571_g1_i1\|m.26330 | 5.885 | 4.221 | 6.256 |
| ***UGT66*** | c93729_g1_i2\|m.26660 | 14.662 | 14.455 | 36.433 |
| ***UGT67(UGT86D1)*** | c93954_g1_i2\|m.27173 | 102.777 | 107.398 | 3.214 |
| ***UGT68*** | c94120_g2_i1\|m.27706 | 1.429 | 0.688 | 1.538 |
| ***UGT69*** | c94157_g1_i1\|m.27807 | 19.843 | 22.709 | 29.344 |
| ***UGT70*** | c94171_g1_i1\|m.27837 | 15.656 | 4.971 | 0.653 |
| ***UGT71*** | c94563_g1_i1\|m.28866 | 5.282 | 2.991 | 4.357 |
| ***UGT72*** | c94823_g1_i1\|m.29476 | 0.849 | 2.095 | 19.583 |
| ***UGT73*** | c94880_g1_i2\|m.29679 | 3.305 | 8.536 | 0 |
| ***UGT74*** | c95291_g3_i1\|m.30951 | 0.301 | 2.418 | 16.18 |
| ***UGT75*** | c95355_g1_i2\|m.31096 | 72.226 | 73.35 | 1.435 |
| ***UGT76*** | c95668_g1_i1\|m.32040 | 58.234 | 44.575 | 81.33 |
| ***UGT77*** | c95803_g1_i1\|m.32401 | 9.748 | 8.879 | 0.945 |
| ***UGT78*** | c96095_g2_i1\|m.33327 | 15.376 | 14.893 | 7.269 |
| ***UGT79*** | c96289_g2_i1\|m.33956 | 11.133 | 5.638 | 12.898 |
| ***UGT80*** | c96499_g1_i1\|m.34699 | 22.166 | 3.147 | 13.774 |
| ***UGT81*** | c96559_g2_i1\|m.34904 | 5.427 | 1.449 | 6.573 |
| ***UGT82*** | c96577_g1_i1\|m.34982 | 11.792 | 16.54 | 119.748 |
| ***UGT83*** | c96727_g1_i1\|m.35458 | 10.262 | 9.421 | 4.571 |
| ***UGT84*** | c96982_g1_i1\|m.36437 | 1.988 | 1.845 | 0.567 |
| ***UGT85*** | c96984_g2_i3\|m.36462 | 6.242 | 7.66 | 9.143 |
| ***UGT86(UGT71H4)*** | c97342_g1_i1\|m.37893 | 343.205 | 98.3 | 0.653 |
| ***UGT87*** | c97425_g1_i3\|m.38294 | 1.909 | 3.2 | 2.148 |
| ***UGT88*** | c97448_g2_i1\|m.38432 | 13.21 | 17.144 | 2.75 |
| ***UGT89(UGT85K18)*** | c97893_g2_i1\|m.40407 | 198.352 | 209.502 | 47.853 |
| ***UGT90*** | c97931_g3_i1\|m.40589 | 5.07 | 13.507 | 2.475 |
| ***UGT91*** | c98038_g2_i4\|m.41121 | 12.719 | 9.432 | 17.211 |
| ***UGT92*** | c98240_g2_i1\|m.41949 | 18.682 | 11.537 | 9.77 |
| ***UGT93*** | c98330_g1_i3\|m.42477 | 8.71 | 11.735 | 9.555 |
| ***UGT94*** | c98410_g12_i1\|m.42854 | 16.728 | 17.321 | 0.808 |
| ***UGT95*** | c98493_g1_i2\|m.43271 | 1.753 | 0 | 19.299 |
| ***UGT96*** | c98532_g2_i2\|m.43454 | 7.839 | 7.598 | 8.421 |
| ***UGT97*** | c98605_g1_i1\|m.43739 | 410.328 | 502.389 | 7.08 |
| ***UGT98*** | c98818_g1_i1\|m.44721 | 1.373 | 0.948 | 9.22 |
| ***UGT99*** | c99091_g1_i1\|m.45972 | 303.452 | 38.79 | 20.966 |
| ***UGT100*** | c99254_g1_i1\|m.46852 | 60.958 | 85.793 | 34.955 |
| ***UGT101*** | c99828_g2_i1\|m.50055 | 5.84 | 6.347 | 3.368 |
| ***UGT102*** | c36215_g1_i1\|m.2929 | 0.78 | 0.27 | 0 |
