# Supplementary material for: Transcriptome-Guided Mining of Genes Involved in Crocin Biosynthesis
Source: Front Plant Sci. 2017 Apr 11;8:518. doi: 10.3389/fpls.2017.00518 (PMC5387100; doi:10.3389/fpls.2017.00518)
Supplement: Supplementary Table 7 — The physicochemical property of CCDs, ALDHs, and UGTs proteins, which used in phylogenetic analysis. [file Table7.DOCX]

**Supplemental Table 7. The Physicochemical Property of CCDs, ALDHs and UGTs Proteins, which used in Phylogenetic Analysis.**

| Gene Name | Protein（aa） | Mw（Da） | p*I* |
| --- | --- | --- | --- |
| AtCCD1 *Arabidopsis thaliana* | 538 | 60908.11 | 6.05 |
| CmCCD1 *Cucumis melo* | 543 | 60837.74 | 6.18 |
| MtCCD1 *Medicago truncatula* | 540 | 60930.81 | 6.30 |
| CmCCD4a *Chrysanthemum morifolium* | 599 | 66881.88 | 8.24 |
| AtCCD4 *Arabidopsis thaliana* | 595 | 65601.73 | 6.42 |
| CitCCD4 *Citrus unshiu* | 563 | 63060.24 | 8.34 |
| AtCCD7 *Arabidopsis thaliana* | 629 | 70850.30 | 6.30 |
| AtCCD8 *Arabidopsis thaliana* | 570 | 63956.91 | 6.65 |
| AtNCED3 *Arabidopsis thaliana* | 599 | 65856.66 | 5.90 |
| AtNCED6 *Arabidopsis thaliana* | 577 | 63821.02 | 5.92 |
| CCD1 *Gardenia jasminoides* | 354 | 40264.85 | 7.68 |
| AtNCED5 *Arabidopsis thaliana* | 589 | 65337.05 | 5.60 |
| CCD4a *Gardenia jasminoides* | 664 | 72925.50 | 6.42 |
| NCED1 *Gardenia jasminoides* | 261 | 29214.39 | 5.29 |
| NCED2 *Gardenia jasminoides* | 640 | 70524.32 | 7.00 |
| CsCCD2 *Crocus sativus* | 562 | 63298.50 | 6.59 |
| ALDH12 *Gardenia jasminoides* | 503 | 54640.84 | 6.11 |
| ALDH14 *Gardenia jasminoides* | 537 | 58522.86 | 6.25 |
| CaALDH1 *Capsicum annuum* | 500 | 54870.15 | 5.89 |
| REF1 *Brassica napus* | 501 | 54295.31 | 6.11 |
| AtALDH2B7 *Arabidopsis thaliana* | 534 | 58152.57 | 6.87 |
| AtALDH2C4 *Arabidopsis thaliana* | 501 | 54360.29 | 5.47 |
| BoBADH *Bixa orellana* | 504 | 54559.57 | 6.28 |
| BALDH *Antirrhinum majus* | 534 | 58189.72 | 6.45 |
| AaALDH1 *Artemisia annua* | 499 | 53799.64 | 6.85 |
| Zmrf2 (ALDH2B2) *Zea mays* | 549 | 59446.20 | 6.69 |
| UGT60 *Gardenia jasminoides* | 446 | 50291.02 | 5.31 |
| UGT67 *Gardenia jasminoides* | 493 | 55671.79 | 6.40 |
| UGT89 *Gardenia jasminoides* | 458 | 51760.96 | 5.29 |
| UGT86 *Gardenia jasminoides* | 477 | 53175.93 | 6.03 |
| UGT73K1 *Medicago truncatula* | 484 | 53925.78 | 6.11 |
| UGT75L6 *Gardenia jasminoides* | 474 | 53008.20 | 5.02 |
| UGT94E5 *Gardenia jasminoides* | 444 | 49798.34 | 5.44 |
| UGT73C6 *Arabidopsis thaliana* | 495 | 55928.59 | 5.18 |
| Gt5GT7 *Gentiana triflora* | 504 | 55290.79 | 5.44 |
| UGT73B2 *Arabidopsis thaliana* | 483 | 54215.01 | 5.92 |
| ZOG1 *Phaseolus lunatus* | 459 | 51411.54 | 6.23 |
| UGTPg45 *Panax ginseng* | 457 | 51152.90 | 5.25 |
| UGTPg29 *Panax ginseng* | 442 | 49147.37 | 5.70 |
| UGT1 *Panax ginseng* | 475 | 53374.43 | 5.25 |
| ZOX1 *Phaseolus vulgaris* | 454 | 50971.94 | 5.61 |
| VLOGT1 *Vitis labrusca* | 448 | 49841.00 | 5.23 |
| VLOGT2 *Vitis labrusca* | 447 | 49713.82 | 5.41 |
| VLOGT3 *Vitis labrusca* | 464 | 51053.18 | 5.18 |
| UGTCs2 *Crocus sativus* | 460 | 50722.01 | 8.46 |
| CsGT45 *Crocus sativus* | 497 | 54972.01 | 5.30 |
| AdGT4 *Actinidia deliciosa* | 484 | 53905.09 | 5.41 |
| GAME2 *Solanum lycopersicum* | 482 | 54225.79 | 5.80 |
| CrUGT8 *Catharanthus roseus* | 482 | 54244.56 | 5.93 |
